# Mechanical analysis of non-Newtonian nanofluid past a thin needle with dipole effect and entropic characteristics

**DOI:** 10.1038/s41598-021-98128-z

**Published:** 2021-09-29

**Authors:** Muhammad Ramzan, Noor Saeed Khan, Poom Kumam

**Affiliations:** 1 KMUTTFixed Point Research Laboratory, Room SCL 802 Fixed Point Laboratory, Science Laboratory Building, Department of Mathematics, Faculty of Science, King Mongkut’s University of Technology Thonburi (KMUTT), Bangkok, 10140 Thailand; 2 Center of Excellence in Theoretical and Computational Science (TaCS-CoE), Science Laboratory Building, Faculty of Science, King Mongkut’s University of Technology Thonburi (KMUTT), 126 Pracha-Uthit Road, Bang Mod, Thung Khru, Bangkok, 10140 Thailand; 3 Department of Medical Research, China Medical University Hospital, China Medical University, Taichung, 40402 Taiwan; 4 Department of Mathematics, Division of Science and Technology, University of Education, Lahore, 54000 Pakistan

**Keywords:** Engineering, Materials science, Mathematics and computing, Nanoscience and technology, Physics

## Abstract

The study concerns with the mechanical characteristics of heat and mass transfer flow of a second grade nanofluid as well as gyrotatic microorganism motion past a thin needle with dipole effect, entropy generation, thermal radiation, Arrhenius activation energy and binar chemical reaction. The governing equations and boundary conditions are simplified by the use of suitable similarity transformations. Homotopy analysis method is implemented to obtain the series solution of non-linear ordinary differential equations. Physical behaviors of heat and mass transfer flow with gyrotatic microorganisms and entropy generation are investigated through the embedded parameters. The nanofluid velocity is enhanced for higher values of the ferromagnetic parameter, local Grashof number, bioconvection Rayleigh number and radiation parameter. The Reynolds number, radiation parameter and Eckert number decrease the nanofluid temperature. The entropy generation is increased with the enhancement of radiation parameter, Eckert number, Lewis number, temperature difference parameter, dimensionless constant parameter, Curie temperature, Prandtl number and concentration difference parameter.

## Introduction

Many researchers and scientists have scrutinized the flow and heat transfer using a moving thin needle because of its importance in a variety of scientific and industrial applications. In this framework, several flow conditions have been considered. Heat transfer is utilized in a variety of specific applications, including metals, polymers, ceramics, anemometers, microscale cooling devices and hot wire for heat removal. Therefore, different researchers and scientists discussed the heat transfer in their research area. Song et al.^1^ considered the study of heat transfer in peripheral air vaporizer used in a refrigerated storage tank in which they obtained that the presence of porous foam increased the heat rate transmission. Doranehgard et al.^2^ examined the simulation of natural gas in oil-saturated tight porous medium. Barnoon et al.^3^ explored the production of hydrogen through the propane steam reforming inside the reactor that consists of shell and tube heat exchanger. Moravej et al.^4^ detected the silver water nanofluid in a hemispherical three-dimensional solar collector operating system. Barnoon et al.^5^ explained the thermal management in biological tissue to prevent tissue destruction during a local heating process. Doranehgard and Dehghanpour^6^ described the numerical and analytical study for the quantification of diffusive and convective transport during the desolvation of $CO_{2}$ in oil. Barnoon and Ashkiyan^7^ used the finite element method for the description of mathematical modeling of heat dispersion and eradication of tumor tissues in the liver utilizing microwaves. The influence of low to medium frequencies and low to medium power within the tissue is explored using a tiny antenna coupled to a power supply. Barnoon et al.^8^ scrutinized the behavior of heat transfer rate, MHD and porous medium over the mixed convection flow of ferrofluid through the rotating cylinder by using three distinct temperature cases with local thermal equilibrium and non-equilibrium. Barnoon et al.^9^ inspected the heat transfer and fluid flow by the existence of magnetic field along with the porous media and rotating circular obstacles. They identified that increasing the volume fraction improves the heat transfer rate. Barnoon et al.^10^ scrutinized the thermal performance, entropy generation and heat transfer of a two-phase nanofluid model embedded in a conical vane inside a circular porous channel and they observed that the heat transfer rate is enhanced with the drop of pressure. Shahsavar et al^11^ pointed out the investigations of heat transfer, hydrothermal and entropy generation features in a naturally cooled eccentric horizontal annulus from the perspective of first-law and second-law of thermodynamics. Shahsavar et al.^12^ employed the first and second law of thermodynamics to check the influence of water-silver biological nanofluid over the performance of newly designed heat sink in a porous media. Barnoon et al.^13^ discussed the heat transfer and nanofluid model in a permeable enclosure with thermal radiation and two-phase natural convection impacts toward a porous cavity. Barnoon and Toghraie^14^ analyzed the numerical investigations of heat transfer and laminar flow of non-Newtonian nanofluid toward the porous media. Various phenomena shown through the different differential equations have been existed and can be found in the references^15–18^.

Over the last few years, the scientists and researchers have considerable interest in non-Newtonian fluid because of their extensive applications in engineering and industries. Such type of fluid is used in oil store building, foods stuffs, material handling, synthetic processes, electronic packaging, enhanced oil recovery, polymer processing etc^19–21^. Additionally, on the base of physical applications in industries and engineering a lot of researchers have broadly studied the non-Newtonian fluid. Bilal and Urva^22^ observed the presence of activation energy and variable viscosity in the non-Newtonian fluid toward the thin needle. In their study, they used MATLAB bvp4c built-in function for the solution of the ordinary differential equations and found that the fluid concentration becomes higher for activation energy. Khan et al.^23^ explained the non-Newtonian Odroyd-B fluid with activation energy and non-linear thermal radiation significance over the rotating disk and initiated that with the enhancement of radiation parameter, the transfer of heat becomes stronger. Gaffar et al.^24^ detected Jefferrey’s non-Newtonian fluid over the semi-infinite vertical plate with the effect of heat generation and noticed the different applications of their research in chemical and mechanical engineering such as nuclear waste simulation and polymeric manufacturing process. Waqas et al.^25^ studied the bioconvection non-Newtonian fluid with the motile microorganisms past a rotating disk. With the help of similarity transformations, they obtained the higher order ordinary differential equations of their model from the leading partial differential equations and also found some useful applications of rotating disks in engineerings such as crystal growth, lubrication, oceanography, computer storage devices and rotating machinery.

The magnetic dipole effect is an important phenomenon in engineering and industrial applications therefore, the researchers and scientists mostly use the magnetic dipole effect in their research work. Alshomrani^26^ investigated the magnetic dipole effect on the flow of nanofluid along a stretched cylinder with gyrotactic microorganisms in a stratified medium. Zeeshan and Majeed^27^ established a Jeffery fluid flow mathematical model with the existence of magnetic dipole and heat transfer analysis due to the stretching surface through the applications of shooting method along with R-K method and obtained a series solution of the problem. Majeed et al.^28^ have investigated the magnetic dipole effect in a Maxwell ferrofluid in the presence of chemical reaction and they noticed that the thickness of the boundary layer and velocity of the fluid are reduced when the Maxwell parameter rises. Khan et al.^29^ calculated the thermal radiation behavior through the Williamson nanofluid with the manifestation of magnetic dipole effect and claimed that as the values of thermophoresis and Brownian motion parameters are changed, the fluid temperature falls. Hayat et al.^30^ discussed the dipole effect in a ferromagnetic second grade fluid toward the porous media and found some important results of skin friction, Nusselt number, velocity and temperature of the fluid in a graphical and tabular form.

The suspension of nanometer size particles in the base fluid is called nanofluid. Metals, their oxides, carbides, and carbon nanotubes are the main components of nanoparticles utilized in nanofluids. Nanofluids are beneficial in a variety of applications, including microelectronics, fuel cells, pharmaceutical procedures, heating systems, temperature controllers, gas vented from chimneys, dispersion of heat, machine for propelled cross breening and so on^31,32^. Based on the importance of nanofluid many experimental and theoretical observations are being executed by different researchers. Waqas et al.^33^ analyzed the non-Newtonian nanofluid with the radiation and dipole impact. Most of the researchers use the nanofluid with zero mass flux conditions or convective heat conditions but according to the physical phenomena to make problem more optimistic and realistic they solved their model with both convective heat conditions and zero mass flux conditions and concluded that the boundary layer thickness is increased for the radiation effect. Akbari et al.^34^ evaluated the heat transfer effect in a non-Newtonian nanofluid and their results show that the heat transfer rate increases with the increase of nanoparticles of solid volume fraction. Naz et al.^35^ reported the behavior of activation energy in the numerical solution of the non-Newtonian nanofluid via shooting technique and scrutinized that the nanoparticles volume friction is improved for the enhancement of activation energy. Shafique et al.^36^ examined the influence of buoyancy impact through the bioconvection second-grade nanofluid in which they studied that the fluid concentration is affected by the increase of buoyancy. Ali et al.^37^ pointed out the study of heat source/sink and gyrotactic micro-organisms in a three-dimensional Maxwell nanofluid on a stretching surface and their numerical solution explained that the bioconvection Lewis number $Lb$ and bioconvection Peclet number $Pe$ upsurge the motile micro-organisms density. Khan et al.^38^ introduced the mathematical model of Casson nanofluid between two stretching disks along with the influence of entropy generation. For the numerically investigation of the model, they applied the shooting technique. Ashraf et al.^39^ modeled a problem of non-Newtonian nanofluid with the combined occurrence of Brownian motion and thermophoresis impacts. 

In medical research, the bioconvection has been studied for over a couple of centuries. The typical upwardly swimming microorganisms, which are slightly denser than water in suspensions, cause this phenomenon. When the upper surface of the suspension becomes too thick due to microorganisms accumulation, the suspension becomes unstable, causing microorganisms to tumble and bio-convection currents to form. Despite the fact that comprehensive mathematical studies on bioconvection phenomena have been published, the importance of this phenomenon has attracted the attention of medical engineers in bio-diesel fuels, bio reactors and fuel cell technology. Bhatti et al.^40^ delivered the study of motile gyrotactic microorganisms in a non-Newtonian blood-based nanofluid by a cylindrical co-ordinate system and showed that throughout the whole flow channel, the behavior of the fluid flow remains same in non-tapered, converging and diverging arteries. Sohail et al.^41^ interpreted the non-Newtonian fluid flow with gyrotactic microorganisms in the presence of mass and heat transport behavior under the nonlinear stretching surface. For the physical formulation of the model, they employed the boundary layer theory in terms of partial differential equations and enhancement in reaction is noted in the profile of the motile density for Peclet number. Pal and Mondal^42^ presented the influence of gyrotactic microorganisms on a bioconvection nanofluid with thermal radiation and chemical reaction effects. In this article, they expressed that the chemical reaction shows the increasing effect over the nanoparticles concentration but decreasing behavior is observed for the thermal radiation. Haq et al.^43^ proposed the existence of gyrotactic microorganisms in a mathematical modelling of Williamson fluid in the prevalence of activation energy past a porous surface of cylinder. Gyrotactic microorganisms are used as controlling agents for the random movement of nanoparticles that are suspended. Khan et al.^44^ disclosed the Walter-B fluid subjected to gyrotactic microorganisms along with the heat transport phenomena by examining the graphical and tabular representation of the problem by built-in shooting technique. Nima et al.^45^ expressed another study of gyrotactic microorganisms in a non-Newtonian fluid over the vertical plate that are embedded in a porous medium where they determined that the non-Newtonian fluid temperature and velocity show the opposite trend for mixed convection parameter.

For the irreversible process, the entropy always increases. Heating and cooling are common occurrence in a variety of engineering and industrial processes, most notably in combustion engines, refrigerators, and air conditioners etc. Entropy generation is used to calculate the efficiency of such energy and electronic devices, and irreversibilities reduce the performance of such devices. Entropy generation and Bejan number can improve the efficiency of any system. Quantity and quality of energy are very essential components in the development and manufacturing of industrial products. To determine the degree and quality of dissipation of energy during a process, the second law of thermodynmics allocates the necessary tools. For assessing the energy quality, the most powerfull tool which is known as a entropy is used. The energy is lost during the transmisison of energy into the usefull work according to the second law of thermodynamics which affects the efficiency of energy conversion devices. It is noted form the definition that both enetropy generation and energy destruction are proportional to each other. As a result, the amount of available energy in a system decreases when the entropy is formed. Decreasing the amount of entropy generation improves the thermal system performance. That’s why during the thermodynamic process entropy generation is most significant tool. Several experimental and theoretical investigations for entropy generation are being conducted by different researchers. Shojaeian and Kosar^46^ performed a study on a non-Newtonian fluid with the properties of entropy generation and heat transfer analysis through the parallel plates along with the slip conditions and discussed that by the existence of slip conditions, the entropy generation is reduced. Shahsavar et al.^47^ considered a two-phase ferrofluid model in a minichannel with the presence of entropy generation and magnetic dipole effect where the global total entropy generation rate is unaffected by the location of the dipole which is not exists in case of two and three dipoles. Khan et al.^48^ offered the aspect of activation energy and chemical reaction in a non-Newtonian nanofluid flow over the stretching surface and obtained results which described that the activation energy decreases the concentration of the fluid. By the implementation of shooting technique along with Runge-Kutta method, Kumar et al.^49^ made a debate on an entropy generation in a Williamson nanofluid in the presence of thermal radiation in which they discoursed that the fluid temperature becomes higher with the rising of radiation parameter and entropy generation shows the decreasing trend for concentration and temperature difference parameter.

The importance of numerous industrial and technological applications such as microscale cooling devices, hot wire anemometers and microstructure electronic devices prompted the researchers to investigate flow and heat transfer through a moving thin needle. The moving thin needle is being considered as a physical model of the present study. Additionally, a thin needle is identified as a parabolic rotation around its axis direction. In the present study, the non-Newtonian second grade nanofluid with gyrotactic microorganisms are discussed in heat and mass transfer flow. The magnetic dipole effect, activation energy, radiation effect and entropy analysis are also discussed through the use of homotopy analysis method. The influence of distinct quantities is discoursed in the graphical from.

## Methods

### Basic equations

**Figure 1:**
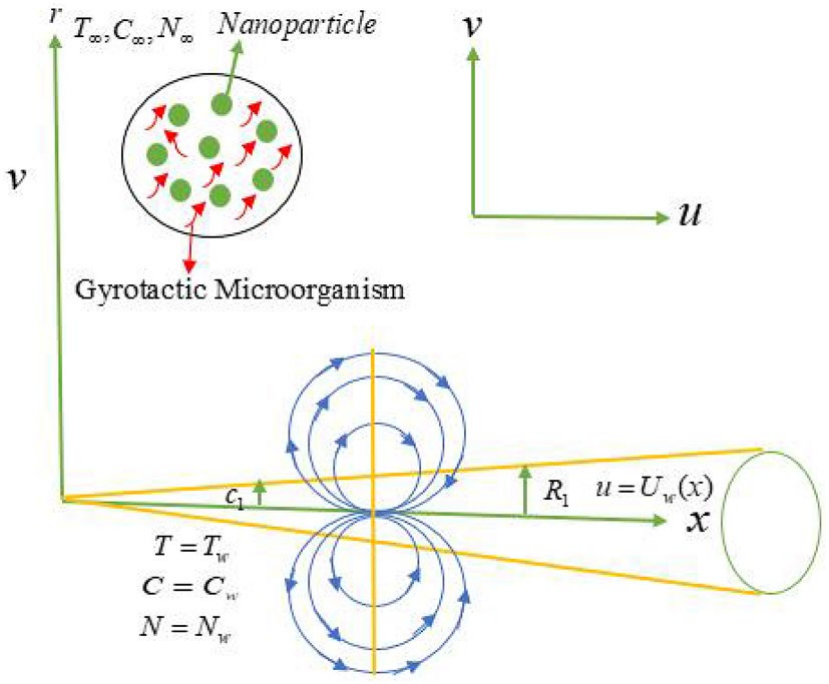
Geometry of the problem.

Consider the steady, laminar and two-dimensional non-Newtonian flow of the second grade nanofluid toward the moving thin needle. The radius of the thin needle is $r ={R_{1}} =\left(\frac{\nu a x }{U}\right)^{\frac{1}{2}}$ where *r* is the radial coordinate and *x* is the axial coordinate as shown in Fig. 1. Here $\nu$ is the kinematic viscosity, size of the needle is $a$ and the needle is moving horizontally with a flow speed $U_{w}$. The needle’s thickness is also considered to be comparable to or less than that of the temperature and momentum boundary layer, but the curvature in the transverse direction has a substantial impact. The pressure difference across the needle is insignificant. The magnetic dipole and thermal radiation effects are taken into account. In the concentration equation the Arrhenius activation energy and binary chemical reaction are observed. Furthermore, in the flow analysis, the gyrotactic microorganisms and entropy generation behaviors are considered. Additionally, the needle surface is kept constant at the wall temperature, nanoparticles concentration and motile gyrotactic microorganisms concentration ${T_{w}}$, ${C_{w}}$ and ${N_{w}}$ which are higher than from the ambient temperature ${T_{\infty }}$, ambient nanoparticles concentration ${C_{\infty }}$ and ambient motile gyrotactic microorganisms concentration ${N_{\infty }}$.

The leading equations for the present model are^22,26^

1 $$\begin{aligned}{}&\frac{\partial (ru)}{\partial x} + \frac{\partial ( rv)}{\partial r}=0, \end{aligned}$$

2 $$\begin{aligned} u \frac{\partial u}{\partial x} + v \frac{\partial u}{\partial r} &= \nu \left(\frac{1}{r}\frac{\partial u}{\partial r} + \frac{\partial ^2 u}{\partial r^{2}}\right) + \frac{\alpha _1}{\rho }\left( v \frac{\partial ^{3} u}{\partial r^{3}} + u \frac{\partial ^{3} u}{\partial x \partial r^{2}} +\frac{\partial u}{\partial x}\frac{\partial ^{2} u}{\partial r^{2}} - \frac{\partial u}{\partial r}\frac{\partial ^{2} v}{\partial r^{2}} + \frac{1}{r}\left(v \frac{\partial ^{2} u}{\partial r^{2}}+u \frac{\partial ^{2} u}{\partial x \partial r} + \frac{\partial u}{\partial x}\frac{\partial u}{\partial r} - \frac{\partial u}{\partial r}\frac{\partial v}{\partial r} \right)\right)\nonumber \\&\quad +\frac{\mu _0}{\rho }M\frac{\partial H}{\partial x}+\left((1-C_{\infty })(T-T_{\infty }){\beta ^{*}}\rho _{f}-(\rho _{P}-\rho _{f})(C-C_{\infty })-\gamma (\rho _{m}-\rho _{f})(N-N_{\infty })\right) g , \end{aligned}$$

3 $$\begin{aligned} u \frac{\partial T}{\partial x} + v \frac{\partial T}{\partial r}&=\frac{K}{\rho {C_{P}}}\left(1+ \frac{16\sigma _{1}{T_{\infty }^{3}}}{3k_{e}K}\right) \frac{1}{r}\frac{\partial }{\partial r}\left({r}\frac{\partial T}{\partial r}\right) +\tau \left({D_{B}}\frac{\partial T}{\partial r}\frac{\partial C}{\partial r} + \frac{D_{T}}{T_{\infty }}\left(\frac{\partial T}{\partial r}\right)^2\right)\nonumber \\&\quad - \frac{\mu _0}{\rho {C_{P}}}T\frac{\partial M}{\partial T}\left(u \frac{\partial H}{\partial x}+v \frac{\partial H}{\partial r}\right), \end{aligned}$$

4 $$\begin{aligned}{}&u \frac{\partial C}{\partial x} + v \frac{\partial C}{\partial r}=\frac{D_{B}}{r}\frac{\partial }{\partial r}\left({r}\frac{\partial C}{\partial r}\right) + \frac{D_{T}}{T_{\infty }}\frac{1}{r}\frac{\partial }{\partial r}\left({r}\frac{\partial T}{\partial r}\right) - k_{cr}^{2}\left( C-{C_{\infty }}\right)\left(\frac{T}{T_{\infty }}\right)^{n}\exp \left(\frac{- E_{a_{1}}}{\kappa T}\right), \end{aligned}$$

5 $$\begin{aligned}{}& u \frac{\partial N}{\partial x} + v \frac{\partial N}{\partial r} + \frac{ bW_{c}}{(C_{w} - C_{\infty })}{\frac{\partial }{\partial r}\left({N}\frac{\partial C}{\partial r}\right)} = {D_{m}}\frac{1}{r}\frac{\partial }{\partial r}\left({r}\frac{\partial N}{\partial r}\right). \end{aligned}$$

The boundary conditions are

6 $$\begin{aligned}{}&u = U_{w},\quad v = 0,\quad T = T_{w},\quad D_{B}\frac{\partial C}{\partial r}+\frac{D_{T}}{T_{\infty }}{\frac{\partial T}{\partial r}}=0,\quad N = N_{w},\quad {at}\quad r = R_{1}(x), \end{aligned}$$

7 $$\begin{aligned}{}&\quad u \rightarrow 0,\quad T \rightarrow T _{\infty },\quad C \rightarrow C _{\infty },\quad N \rightarrow N _{\infty } \quad{when}\quad r \rightarrow {\infty }, \end{aligned}$$

where *u*(*x, r*), *v*(*x, r*) are used for the velocity components, $\rho$ is the density, the $\rho _{f}$, $\rho _{m}$, $\rho _{P}$ are the densities of base fluid, motile microorganism and nanoparticles, ${\beta ^{*}}$ denotes the coefficient of volumetric volume expansion, $\alpha _{1}$ is used for the second grade fluid parameter, the magnetic permeability is $\mu _{0}$, the magnetization is expressed by $M$, through the unit volume, the ferromagnetic body force is $\frac{\mu _0}{\rho }M\frac{\partial H}{\partial x}$, $H$ is the magnetic field, the fluid thermal diffusivity is denoted by $\alpha$, the nanofluid effective heat capacity ratio is $\tau$, *T* is the nanofluid temperature and *C* is the nanofluid concentration, the motile gyrotactic microorganism density is *N*, $g$ is the acceleration due to gravity. The nanofluid thermal conductivity is *K*, $C_{P}$ is the specific heat at a constant pressure, the radiative heat flux is $q_{r}=-\frac{16}{3}\left(\frac{\sigma _{1}{T_{\infty }}^{3}}{k_{e}}\frac{\partial T}{\partial r}\right)$, here Stefan–Boltzmann constant is $\sigma _{1}$ and the mean adsorption coefficient is $k_{e}$, the chemical reaction constant is $k_{cr}$ and the Brownian diffusion coefficient for microorganism is $D_{m}$. *n* is the fitted rate constant such that (−1 < *n* < 1), $E_{a_{1}}$ is the activation energy in which $a_{1}$ is the positive dimensional constant, $\kappa$ = 8.61 $\times$ 10$^{-5}$eV/K is the Boltzmann constant and $k_{cr}^{2}$ $({C}- {C}_{\infty }) \left(\frac{T}{T_{\infty }}\right)^{n}\exp \left(\frac{- E_{a_{1}}}{\kappa T}\right)$ is the modified Arrhenius term. $b$ is known as chemotaxis constant, $W_{c}$ is used for maximum amount of swimming cell, the Brownian diffusion and thermophoresis diffusion coefficients are denoted by $D_{B}$, $D_{T}$ respectively. Similarity transformations are introduced for the conversion of the Eqs. (2–5) into the dimensionless form as^22,26^

8 $$\begin{aligned} \psi (x, r )=\nu x f ,\zeta =\frac{U{r}^{2}}{\nu {x}},\theta (\zeta ) = \frac{T-T_{\infty }}{T_{w}-T_{\infty }},\phi (\zeta ) = \frac{C-C_{\infty }}{C_{w}-C_{\infty }},\chi (\zeta ) = \frac{N-N_{\infty }}{N_{w}-N_{\infty }}, \end{aligned}$$

where $\zeta$ is the similarity variable, $\psi$ is the stream function and $u =r^{-1}\frac{\partial \psi }{\partial r}$, $v =r^{-1}\frac{\partial \psi }{\partial x}$. The dimensionless velocity, temperature, nanoparticles concentration and motile microorganism concentration are designated by the $f^{\prime } , \theta$, $\phi$ and $\chi$ respectively. With the help of similarity transformations in Eq. (8), the equation of continuity defined in Eq. (1) is identically satisfied and then by using Eq. (8), dimensionless equations from Eqs. (2–5) are obtained as

9 $$\begin{aligned}{} & 2\left(\zeta f ^{\prime \prime \prime } + f ^{\prime \prime }\right) + f f ^{\prime \prime } - \delta \left(2\zeta f f ^{\prime \prime \prime \prime }+2\zeta f ^{\prime } f ^{\prime \prime \prime }+3\zeta f f ^{\prime \prime \prime }+\frac{3}{2} f ^{\prime } f ^{\prime \prime }+4\zeta ^{2} f ^{\prime \prime } f ^{\prime \prime \prime }+4\zeta {f ^{\prime \prime }}^{2}+f f ^{\prime \prime \prime }\right)\nonumber \\&\quad -\frac{\beta }{4}{\frac{\zeta ^{2}}{(\zeta +\gamma _{1})^{4}}}{\theta } + \frac{1}{8} \left(Gr \theta -Nr \phi -Rb \chi\right) =0, \end{aligned}$$

10 $$\begin{aligned}{}&\quad \left(\frac{1+R}{Pr}\right)\left(\zeta {\theta }^{\prime }{\prime }+\theta ^{\prime }\right)+\frac{1}{2} f {\theta }^{\prime }+\zeta Nb \theta ^{\prime }\phi ^{\prime }+\zeta Nt {\theta ^{\prime }}^{2} +\zeta \beta Ec (\theta -\epsilon )\left(\frac{\zeta }{(\zeta +\gamma _{1})^{4}} f ^{\prime }-\frac{1}{2} Re \frac{-\zeta f ^{\prime }+f }{(\zeta +\gamma _{1})^{3}} +\zeta \frac{-\zeta f ^{\prime }+f }{(\zeta +\gamma _{1})^{5}} \right)=0, \end{aligned}$$

11 $$\begin{aligned}{}&\quad 2(\zeta \phi ^{\prime \prime }+\phi ^{\prime })+2\frac{Nt}{Nb}\left(\zeta \theta ^{\prime \prime }+\theta ^{\prime }\right)+ {Le} f \phi ^{\prime } -\frac{1}{2} {Le}\Gamma (1+\Omega _{1}\theta )^{n}\phi \exp \left(\frac{-E}{1 + \Omega _{1}\theta }\right) = 0, \end{aligned}$$

12 $$\begin{aligned}{}&\quad 2\zeta \left(\chi ^{\prime \prime }- {Pe}\chi \phi ^{\prime \prime }- {Pe}\lambda \phi ^{\prime \prime }- {Pe}\chi ^{\prime }\phi ^{\prime }\right) +\chi ^{\prime }(1+ {Lb} f ) = 0, \end{aligned}$$

13 $$\begin{aligned}{}&\quad f = \zeta \frac{\epsilon }{2},\quad {f^{\prime }} = \frac{\epsilon }{2},\quad \theta = 1,\quad Nb \phi ^{\prime }+Nt \theta ^{\prime }=0, \chi = 1,\quad at \quad \zeta = 1, \end{aligned}$$

14 $$\begin{aligned}{}&\quad f^{\prime } = 0,\quad \theta = 0,\quad \phi = 0,\quad \chi = 0\quad at\quad \zeta = \infty , \end{aligned}$$

where ($^{\prime }$) is used for differentiation with respect to $\zeta$. The elasticity parameter is $\delta =\frac{\alpha _{1}{U}}{\rho \nu x }$, the non-dimensional distance between the origin and center of magnetic dipole is represented by $\gamma _{1}=\frac{Urc_{1}}{\nu x }$, the ferromagnetic parameter is $\beta =\frac{\rho \gamma \mu _{0}k^*(T_{\infty }-T_{w})}{\mu ^{2}}$, $Ec =\frac{U^{2}}{C_{P}(T_{w}-T_{\infty })}$ is the Eckert number, $\epsilon =\frac{T_{\infty }}{T_{\infty }-T_{w}}$ is the Curie temperature, the local Grashof number is $Gr =\frac{\rho _{f}{\beta ^{*}} g x (1-C_{\infty })(T_{w}-T_{\infty })}{U ^{2}}$, $Nr =\frac{g x (\rho _{P}-\rho _{f})(C_{w}-C_{\infty })}{U^{2}}$ is the buoyancy ratio parameter, the bioconvection Rayleigh number is $Rb =\frac{\gamma g x (\rho _{m}-\rho _{f})(N_{w}-N_{\infty })}{U^{2}}$, radiation parameter is specified by $R =\frac{16\sigma _{1}{T_{\infty }^{3}}}{3k_{e}K}$, the Prandtl number is $Pr =\frac{\nu }{\alpha }$, the Brownian motion parameter is manifested by $Nb =\frac{\tau D_{B} (C_{w}-C_{\infty })}{\nu }$, thermophoresis parameter is symbolized by $Nt =\frac{\tau D_{T} (T_{w}-T_{\infty })}{\nu {T_{\infty }}}$, the Lewis number is $Le =\frac{\nu }{D_{B}}$, $\Gamma =\frac{k_{cr}^{2} x }{U}$ is the binary chemical reaction parameter, the temperature difference parameter is signified by $\Omega _{1}=\frac{T_{w}-T_{\infty }}{T_{\infty }}$, the activation energy parameter, bioconvection Lewis number, bioconvection Peclet number, the bioconvection constant parameter and the Reynolds number are denoted by $E =\frac{Ea_{1}}{k{T_{\infty }}}$, $Lb =\frac{\nu }{D_{m}}$, $Pe =\frac{b{W_{c}}}{D_{m}}$, $\lambda =\frac{N_{\infty }}{N_{w}-N_{\infty }}$ and $Re =\frac{Ux}{\nu }$ respectively.

Now, it is interesting to discourse some engineering physical quantities such as skin friction coefficient, local Nusselt number, local sherwood number and motile microorganisms as

15 $$\begin{aligned}{}&C_{f}=\frac{\mu }{\rho U^{2}} \left(\frac{\partial u}{\partial r}\right)_{r={a}}=4 R_{e}^{-\frac{1}{2}} {\zeta ^{\frac{1}{2}}} f^{\prime \prime }(\zeta ), \end{aligned}$$

16 $$\begin{aligned}{}&\quad Nu_{x} =\frac{x}{{T_{w}-T_{\infty }}}\left( \frac{\partial T}{\partial r} \right)_{r={a}}=-2 R_{e}^{\frac{1}{2}} {\zeta ^{\frac{1}{2}}}{\theta ^{\prime }}(\zeta ), \end{aligned}$$

17 $$\begin{aligned}{}&\quad Sh_{x} =\frac{-x}{C_{w}-C_{\infty }}\left( \frac{\partial C}{\partial r} \right)_{r={a}}=-2 R_{e}^{\frac{1}{2}} {\zeta ^{\frac{1}{2}}}{\phi ^{\prime }}(\zeta ), \end{aligned}$$

18 $$\begin{aligned}{}&\quad Nn_{x} =\frac{-x}{N_{w}-N_{\infty }}\left( \frac{\partial N}{\partial r} \right)_{r={a}}=- R_{e}^{\frac{1}{2}} {\chi ^{\prime }}(\zeta ). \end{aligned}$$

## Entropy generation

In two-dimensional flow with the existence of thermal radiation, entropy generation per unit volume for incompressible non-Newtonian fluid in cylindrical coordinate system is

19 $$\begin{aligned} E^{\prime \prime \prime }_{gen} =\frac{K}{T^{2}}\left(\frac{\partial T}{\partial r}\right)^{2}+\frac{K}{T^{2}}\begin{bmatrix} \frac{16\sigma _{1}{T_{\infty }^{3}}}{3k_{e}K}\end{bmatrix}\left(\frac{\partial T}{\partial r}\right)^{2}+\frac{\mu }{T}\left(\frac{\partial u}{\partial r}\right)^{2}+\frac{RD}{C_{\infty }}\begin{bmatrix}\left(\frac{\partial C}{\partial r}\right)^{2}+\left(\frac{\partial C}{\partial x}\right)^{2}\end{bmatrix}+\frac{RD}{T_{\infty }}\begin{bmatrix}{\frac{\partial T}{\partial x}}{\frac{\partial C}{\partial x}}+{\frac{\partial T}{\partial r}\frac{\partial C}{\partial r}}\end{bmatrix}. \end{aligned}$$

In Eq. (19), the first term shows the production of entropy because of heat transfer, second term shows the entropy production due to the friction of the fluid, third term is entropy generation due to the viscous dissipation and the last term is used for entropy generation due to the diffusion effects. For characteristic entropy generation, it is defined that

20 $$\begin{aligned} E^{\prime \prime \prime }_{0} =\frac{4 {K} u }{\nu {x}}. \end{aligned}$$

The dimensionless form of the entropy generation is now achieved by using the specified similarity transformations in Eq. (8) as

21 $$\begin{aligned}{}&N_{G}(\zeta ) = \frac{E^{\prime \prime \prime }_{gen}}{E^{\prime \prime \prime }_{0}}. \end{aligned}$$

22 $$\begin{aligned}{}&\quad N_{G}(\zeta ) =\frac{\zeta {\theta ^{\prime }}^{2}}{(\theta -\epsilon )^{2}}(1+R)+ 4\frac{Ec }{Pr}\frac{\zeta {{f}^{\prime \prime }}^{2}}{(\theta -\epsilon )}+\left(1+\frac{A}{4}\right) L \Omega _{1}\zeta {\theta ^{\prime }}{\phi ^{\prime }}+\left(\zeta +\frac{A}{4}\right) L {\alpha _{2}}{\phi ^{\prime }}, \end{aligned}$$

where $N_{G}$ is the non-dimensional entropy generation, the diffusive paramter is $L =\frac{RD (C_{w}-C_{\infty })}{K}$, $A =\frac{r^{2}}{x^{2}}$ is the dimensionless constant parameter and the nanoparticles concentration difference is indicated by $\alpha _{2}=\frac{C_{w}-C_{\infty }}{C_{\infty }}$.

## Solution of the problem

Homotopy analysis technique is employed for the analytical examination of the present study. For this investigation, computer-based programming software in Mathematics 10 is used. The HAM approach is adopted to address the problem because it provides the following advantages.

1. The present method is simulated for the accurate response without linearization and discretization of the nonlinear differential equations.
2. This method can be used with systems that have smaller or larger natural parameters.
3. Convergent solution to the model is gained with the help of this technique.
4. This approach is linear and does not require any base functions.

The initial guesses and linear operators are taken as

23 $$\begin{aligned}{}&f_{0}(\zeta ) = \frac{\epsilon }{2}(1 - \exp (-\zeta )),\theta _{0}(\zeta ) =\exp (-\zeta ),\phi _{0}(\zeta ) = -\frac{Nt}{Nb}\exp (-\zeta ),\chi _{0}(\zeta ) = \exp (-\zeta ), \end{aligned}$$

24 $$\begin{aligned}{}&\quad {\varvec{{L}}}_{f } = f^{\prime \prime \prime }-f^{\prime },\quad {\varvec{{L}}}_{\theta } = \theta ^{\prime \prime }-\theta ,\quad {\varvec{{L}}}_{\phi } = \phi ^{\prime \prime }-\phi ,\quad {\varvec{{L}}}_{\chi } = \chi ^{\prime \prime }-\chi , \end{aligned}$$

then

25 $$\begin{aligned} {\varvec{{L}}}_{f}\left({C_{1}} + C_{2}\exp (\zeta ) + {C_{3}}\exp (-\zeta )\right) = 0, {\varvec{{L}}}_{\theta }\left( {C_{4}}\exp (\zeta ) + {C_{5}}\exp (-\zeta )\right) = 0,\nonumber \\ {\varvec{{L}}}_{\phi }\left( {C_{6}}\exp (\zeta )+ {C_{7}}\exp (-\zeta )\right) = 0, {\varvec{{L}}}_{\chi }\left({C_{8}}\exp (\zeta ) + C_{9}\exp (-\zeta )\right) = 0, \end{aligned}$$

where $C_{i}$(*i* = 1-9) are the arbitrary constants.

### Zeroth order deformation problems

Zeroth order form of the present model is discussed in this section as

26 $$\begin{aligned}{}&(1 - {p}) {{L _{f} }} [{f}(\zeta , {p}) - {f_{0}}(\zeta )] = {p} {h}_{f} {N}_{f} [{f}(\zeta , {p}),\theta (\zeta , p),\phi (\zeta , p),\chi (\zeta , p)], \end{aligned}$$

27 $$\begin{aligned}{}&\quad (1 - {p}) {{L _{\theta } }} [\theta (\zeta , {p}) - \theta _{0}(\zeta )] = {p} {h}_{\theta } {N}_{\theta } [{f}(\zeta , {p}), \theta (\zeta , {p}),\phi (\zeta , {p})], \end{aligned}$$

28 $$\begin{aligned}{}&\quad (1 - {p}) {{L _{\phi } }} [\phi (\zeta , {p}) - \phi _{0}(\zeta )] = {p} {h}_{\phi } {N}_{\phi } [{f}(\zeta , {p}), \theta (\zeta , {p}),\phi (\zeta , {p})], \end{aligned}$$

29 $$\begin{aligned}{}&\quad (1 - {p}) {{L _{\chi } }} [\chi (\zeta , {p}) - \chi _{0}(\zeta )] = {p} {h}_{\chi } {N}_{\chi } [{f}(\zeta , {p}), \theta (\zeta , {p}),\phi (\zeta , {p}), \chi (\zeta , {p})], \end{aligned}$$

where $p$ is the embedding parameter, the non-zero auxiliary parameters are $h _{f}$, $h _{\theta }$, $h _{\phi }$ and $h _{\chi }$. The nonlinear operators in the leading equations of the model are denoted by the $N _{f}$, $N _{\theta }$, $N _{\phi }$ and $N _{\chi }$ and are given as

30 $$\begin{aligned}{}&N _{f}[f(\zeta , p ), \theta (\zeta , p),\phi (\zeta , p),\chi (\zeta , p)] = 2\left(\zeta \frac{\partial ^{3}f(\zeta , p )}{\partial \zeta ^{3}} + \frac{\partial ^{2}f(\zeta , p)}{\partial \zeta ^{2}}\right) + {f(\zeta , {p})}\frac{\partial ^{2}f(\zeta , p)}{\partial \zeta ^{2}}\nonumber \\&\quad -\delta\left( \vphantom{\left(\zeta \frac{\partial ^{2}f(\zeta , p)}{\partial \zeta ^{2}}\right)^{2}}2\zeta {f(\zeta , {p})}\frac{\partial ^{4}f(\zeta , p)}{\partial \zeta ^{4}}+2\zeta \frac{\partial f(\zeta , p)}{\partial \zeta }\frac{\partial ^{3}f(\zeta , p)}{\partial \zeta ^{3}}+3\zeta {f(\zeta , {p})}\frac{\partial ^{3}f(\zeta , p)}{\partial \zeta ^{3}}\right.\\&\quad\left.+\frac{3}{2}\frac{\partial f(\zeta , p)}{\partial \zeta }\frac{\partial ^{2}f(\zeta , p)}{\partial \zeta ^{2}}+4{\zeta }^{2}\frac{\partial ^{2}f(\zeta , p)}{\partial \zeta ^{2}}\frac{\partial ^{3}f(\zeta , p)}{\partial \zeta ^{3}}+4\left(\zeta \frac{\partial ^{2}f(\zeta , p)}{\partial \zeta ^{2}}\right)^{2}\nonumber + {f(\zeta , {p})}\frac{\partial ^{3}f(\zeta , p)}{\partial \zeta ^{3}}\right) \\ &\quad-\frac{\beta }{4}\frac{\zeta ^{2}}{ (\zeta +\gamma _{1})^{4}}\theta (\zeta ,p)+\frac{1}{8} \left(Gr \theta (\zeta ,p)-Nr \phi (\zeta ,p) -Rb \chi (\zeta ,p)\right), \end{aligned}$$

31 $$\begin{aligned}N _{\theta }[f(\zeta , p), \theta (\zeta , p),\phi (\zeta , p)] &=\begin{pmatrix}\frac{1+R}{Pr}\end{pmatrix}\begin{pmatrix}\zeta \frac{\partial ^{2} \theta (\zeta , p)}{\partial \zeta ^{2}}+ \frac{\partial \theta (\zeta , p)}{\partial \zeta }\end{pmatrix}\\ &\quad+\frac{1}{2}{{f(\zeta , {p})}}\frac{\partial \theta (\zeta , p)}{\partial \zeta }+\zeta Nb \frac{\partial \theta (\zeta , p)}{\partial \zeta }\frac{\partial \phi (\zeta , p)}{\partial \zeta }\nonumber \\&\quad +\zeta Nt \begin{pmatrix}\frac{\partial \theta (\zeta , p)}{\partial \zeta }\end{pmatrix}^{2}+\zeta \beta Ec (\theta (\zeta , p)-\epsilon )\\&\quad\begin{pmatrix}{\frac{\zeta }{(\zeta +\gamma _{1})^{4}}}{\frac{\partial f(\zeta , p )}{\partial \zeta }}-\frac{1}{2}{} Re \begin{pmatrix}{\frac{1}{(\zeta +\gamma _{1})^{3}}}\begin{pmatrix}-\zeta \frac{\partial f(\zeta , p )}{\partial \zeta }+{f(\zeta , {p})}\end{pmatrix}\end{pmatrix}\\ +{\frac{\zeta }{(\zeta +\gamma _{1})^{5}}}\begin{pmatrix}-\zeta \frac{\partial f(\zeta , p )}{\partial \zeta }+f (\zeta , p)\end{pmatrix}\end{pmatrix}, \end{aligned}$$

32 $$\begin{aligned} N _{\phi }[f(\zeta , p),\theta (\zeta , p), \phi (\zeta , p)] &= 2\begin{pmatrix}\zeta \frac{\partial ^{2} \phi (\zeta , p)}{\partial \zeta ^{2}}+\frac{\partial \phi (\zeta , p)}{\partial \zeta }\end{pmatrix}+2\frac{Nt}{Nb}\begin{pmatrix}\zeta \frac{\partial ^{2} \theta (\zeta , p)}{\partial \zeta ^{2}}+\frac{\partial \theta (\zeta , p)}{\partial \zeta }\end{pmatrix}+Le {f(\zeta , {p})}\frac{\partial \phi (\zeta , p)}{\partial \zeta }\nonumber \\&\quad -\frac{1}{2}{} Le \Gamma (1+\Omega _{1}\theta (\zeta , p))^{n}\phi (\zeta , p)\exp \begin{pmatrix}\frac{-E}{1 + \Omega _{1}{\theta (\zeta , p)}}\end{pmatrix}, \end{aligned}$$

33 $$\begin{aligned} N _{\chi }[f(\zeta , p), \phi (\zeta , p), \chi (\zeta , p)] &= 2\zeta \begin{pmatrix}\frac{\partial ^{2} \chi (\zeta , p)}{\partial \zeta ^{2}}-Pe \chi (\zeta , p)\frac{\partial ^{2} \phi (\zeta , p)}{\partial \zeta ^{2}}-Pe \lambda \frac{\partial ^{2} \phi (\zeta , p)}{\partial \zeta ^{2}}-Pe \frac{\partial \chi (\zeta , p)}{\partial \zeta }\frac{\partial \phi (\zeta , p)}{\partial \zeta }\end{pmatrix}\nonumber \\&\quad +(1+Lb {f(\zeta , {p})})\frac{\partial \chi (\zeta , p)}{\partial \zeta }, \end{aligned}$$

34 $$\begin{aligned}{}&\quad f (1, p) = \frac{\epsilon }{2},\quad {f^{\prime }} (1, p) =\frac{\epsilon }{2} ,\quad {f^{\prime }} (\infty , p) = 0, \end{aligned}$$

35 $$\begin{aligned}{}&\quad \theta (1, p) = 1,\quad {\theta } (\infty , p) = 0, \end{aligned}$$

36 $$\begin{aligned}{}&\quad \phi ^{\prime } (1, p) = -\frac{Nt}{Nb}\theta ^{\prime },\quad {\phi } (\infty , p) = 0, \end{aligned}$$

37 $$\begin{aligned}{}&\quad \chi (1, p) = 1,\quad {\chi } (\infty , p) = 0. \end{aligned}$$

When *p* = 0 and *p* = 1, then the Eqs. (26–29) give

38 $$\begin{aligned}{}&p = 0 \Rightarrow f (\zeta , 0) = f_{0}(\zeta ) \quad and \quad p = 1 \Rightarrow f (\zeta , 1) = f(\zeta ), \end{aligned}$$

39 $$\begin{aligned}{}&\quad p = 0 \Rightarrow \theta (\zeta , 0) = \theta _{0}(\zeta ) \quad and \quad p = 1 \Rightarrow \theta (\zeta , 1) = \theta (\zeta ), \end{aligned}$$

40 $$\begin{aligned}{}&\quad p = 0 \Rightarrow \phi (\zeta , 0) = \phi _{0}(\zeta ) \quad and \quad p = 1 \Rightarrow \phi (\zeta , 1) = \phi (\zeta ), \end{aligned}$$

41 $$\begin{aligned}{}&\quad p = 0 \Rightarrow \chi (\zeta , 0) = \chi _{0}(\zeta ) \quad and \quad p = 1 \Rightarrow \chi (\zeta , 1) = \chi (\zeta ). \end{aligned}$$

By using Taylor expansion series on Eqs. (38–41), it is obtained that

42 $$\begin{aligned}{}&{f}(\zeta , {p}) = {f_{0}}(\zeta ) + \sum ^{\infty }_{m = 1} {f_{m}}(\zeta ){p^{m}},\quad {f_{m}}(\zeta ) = \frac{1}{m!} \frac{\partial ^{m} {f}(\zeta ,{p})}{\partial \zeta ^{m}}\mid _{p=0}, \end{aligned}$$

43 $$\begin{aligned}{}&\quad \theta (\zeta , {p}) = {\theta _{0}}(\zeta ) + \sum ^{\infty }_{m = 1} {\theta _{m}}(\zeta ){p^{m}},\quad {\theta _{m}}(\zeta ) = \frac{1}{m!} \frac{\partial ^{m} \theta (\zeta , {p})}{\partial \zeta ^{m}}\mid _{p=0}, \end{aligned}$$

44 $$\begin{aligned}{}&\quad \phi (\zeta , {p}) = {\phi _{0}}(\zeta ) + \sum ^{\infty }_{m = 1} {\phi _{m}}(\zeta ){p^{m}},\quad {\phi _{m}}(\zeta ) = \frac{1}{m!} \frac{\partial ^{m} \phi (\zeta , {p})}{\partial \zeta ^{m}}\mid _{p=0}, \end{aligned}$$

45 $$\begin{aligned}{}&\quad \chi (\zeta , {p}) = {\chi _{0}}(\zeta ) + \sum ^{\infty }_{m = 1} {\chi _{m}}(\zeta ){p^{m}},\quad {\chi _{m}}(\zeta ) = \frac{1}{m!} \frac{\partial ^{m} \chi (\zeta , {p})}{\partial \zeta ^{m}}\mid _{p=0}. \end{aligned}$$

From Eqs. (42–45), the convergence of the series is obtained by taking *p* = 1 which implies that

46 $$\begin{aligned}{}&{f}(\zeta ) = {f_{0}}(\zeta ) + \sum ^{\infty }_{m = 1}{f_{m}}(\zeta ) , \end{aligned}$$

47 $$\begin{aligned}{}&\quad \theta (\zeta ) = {\theta _{0}}(\zeta ) + \sum ^{\infty }_{m = 1}{\theta _{m}}(\zeta ) , \end{aligned}$$

48 $$\begin{aligned}{}&\quad \phi (\zeta ) = {\phi _{0}}(\zeta ) + \sum ^{\infty }_{m = 1}{\phi _{m}}(\zeta ) , \end{aligned}$$

49 $$\begin{aligned}{}&\quad \chi (\zeta ) = {\chi _{0}}(\zeta ) + \sum ^{\infty }_{m = 1}{\chi _{m}}(\zeta ) . \end{aligned}$$

### mth order deformation problems

The mth order deformation of the Eqs. (26–29) is

50 $$\begin{aligned}{}&{{L_{f}}}[{f_{m}}(\zeta ) - \eta _{m}{f_{m-1}}(\zeta )] = \hslash _{f} \mathfrak {R}^{f}_{m}(\zeta ), \end{aligned}$$

51 $$\begin{aligned}{}&\quad {{L_{\theta }}}[{\theta _{m}}(\zeta ) - \eta _{m}{\theta _{m-1}}(\zeta )] = \hslash _{\theta } \mathfrak {R}^{\theta }_{m}(\zeta ), \end{aligned}$$

52 $$\begin{aligned}{}&\quad {{L_{\phi }}}[{\phi _{m}}(\zeta ) - \eta _{m}{\phi _{m-1}}(\zeta )] = \hslash _{\phi } \mathfrak {R}^{\phi }_{m}(\zeta ), \end{aligned}$$

53 $$\begin{aligned}{}&\quad {{L_{\chi }}}[{\chi _{m}}(\zeta ) - \eta _{m}{\chi _{m-1}}(\zeta )] = \hslash _{\chi } \mathfrak {R}^{\chi }_{m}(\zeta ), \end{aligned}$$

54 $$\begin{aligned}{}&\quad f_{m}(1) = 0,\quad f^{\prime }_{m}(1) = 0,\quad f^{\prime }_{m}(\infty ) = 0, \end{aligned}$$

55 $$\begin{aligned}{}&\quad \theta _{m}(1) = 0,\quad \theta _{m}(\infty ) = 0, \end{aligned}$$

56 $$\begin{aligned}{}&\quad \phi _{m}(1) = 0,\quad \phi _{m}(\infty ) = 0, \end{aligned}$$

57 $$\begin{aligned}{}&\quad \chi _{m}(1) = 0,\quad \chi _{m}(\infty ) = 0, \end{aligned}$$

where $R _{m}^{f}(\zeta )$, $R _{m}^{\theta }(\zeta )$, $R _{m}^{\phi }(\zeta )$, and $R _{m}^{\chi }(\zeta )$ are defined as

58 $$\begin{aligned}{}&\mathfrak {R}_{m}^{f}(\zeta )=2\begin{pmatrix}\zeta {f^{\prime \prime \prime }_ {m-1}}+f^{\prime \prime }_{m-1}\end{pmatrix}+\sum ^{m - 1}_{k = o}{f_{m - 1 - k} f^{\prime \prime }_{k}}-\delta \begin{bmatrix}2\zeta {\sum ^{m - 1}_{k = o}{f_{m - 1 - k} f^{\prime \prime \prime \prime }_{k}}}+2\zeta {\sum ^{m - 1}_{k = o}{f^{\prime }_{m - 1 - k} f^{\prime \prime \prime }_{k}}}+\\ 3\zeta {\sum ^{m - 1}_{k = o}{f_{m - 1 - k} f^{\prime \prime \prime }_{k}}}+\frac{3}{2}{\sum ^{m - 1}_{k = o}{f^{\prime }_{m - 1 - k} f^{\prime \prime }_{k}}}+\\ 4\zeta ^{2}{\sum ^{m - 1}_{k = o}{f^{\prime \prime }_{m - 1 - k} f^{\prime \prime \prime }_{k}}}+4\zeta {\sum ^{m - 1}_{k = o}{f^{\prime \prime }_{m - 1 - k} f^{\prime \prime }_{k}}}+\\ \sum ^{m - 1}_{k = o}{f_{m - 1 - k} f^{\prime \prime \prime }_{k}}\end{bmatrix}\nonumber \\&\quad -\frac{\beta }{4}{\frac{\zeta ^{2}}{(\zeta +\gamma _{1})^{4}}}\theta _{m}+\frac{1}{8}\begin{pmatrix}{Gr}\theta _{m}-{Nr}\phi _{m}-{Rb}\chi _{m}\end{pmatrix} ,\end{aligned}$$

59 $$\begin{aligned} \mathfrak {R}_{m}^{\theta }(\zeta )&=\begin{pmatrix}\frac{1+R}{Pr}\end{pmatrix} \begin{pmatrix}\zeta {\theta ^{\prime \prime }_{m-1}} +\theta ^{\prime }_{m-1}\end{pmatrix}+\frac{1}{2}\sum ^{m - 1}_{k = o}{f_{m - 1 - k}\theta ^{\prime }_{k}}+\zeta {Nb}\sum ^{m - 1}_{k = o}{\theta ^{\prime }_{m - 1 - k} \phi ^{\prime }_{k}}+\zeta {Nt}\sum ^{m - 1}_{k = o}{\theta ^{\prime }_{m - 1 - k} \theta ^{\prime }_{k}}\nonumber \\&\quad + \zeta \beta {Ec}(\theta _{m}-\epsilon )\begin{pmatrix} {\frac{\zeta }{(\zeta +\gamma _{1})^{4}}}{{f}^{\prime }_{m-1}}- \frac{1}{2}{Re}{\frac{-\zeta {{f}^{\prime }_{m-1}} +{f}_{m}}{(\zeta +\gamma _{1})^{3}}} \\ +\zeta \frac{-\zeta {{f}^{\prime }_{m-1}}+{f}_{m}}{(\zeta +\gamma _{1})^{5}} \end{pmatrix}, \end{aligned}$$

60 $$\begin{aligned}{}&\quad \mathfrak {R}_{m}^{\phi }(\zeta ) =2(\zeta \phi ^{\prime \prime }_{m-1}+\phi ^{\prime }_{m-1})+2\frac{Nt}{Nb}\begin{pmatrix}\zeta \theta ^{\prime \prime }_{m-1}+\theta ^{\prime }_{m-1}\end{pmatrix} +\textit{Le}\sum ^{m - 1}_{k = o}{{f_{m-1-k}}\phi ^{\prime }_{k}} -\frac{1}{2}{} \textit{Le}\Gamma (1+\Omega _{1}\theta _{m})^{n}{\phi _{m}}\exp \begin{pmatrix}\frac{-E}{1 + \Omega _{1}\theta _{m}}\end{pmatrix}, \end{aligned}$$

61 $$\begin{aligned}{}&\quad \mathfrak {R}_{m}^{\chi }(\zeta ) =2\zeta \begin{pmatrix}\chi ^{\prime \prime }_{m-1}-\textit{Pe}\sum ^{m - 1}_{k = o}{\chi _{m-1-k}}\phi ^{\prime \prime }_{k} -\textit{Pe}\lambda {\phi ^{\prime \prime }_{m-1}}-\textit{Pe}\sum ^{m - 1}_{k = o}{\chi ^{\prime }_{m-1-k}\phi ^{\prime }_{k}}\end{pmatrix}+\chi ^{\prime }_{m-1}+ \textit{Lb}\sum ^{m - 1}_{k = o}{{f}_{m-1-k}\chi ^{\prime }_{k}}, \end{aligned}$$

62 $$\begin{aligned} \eta _{m} = \left\{ \begin{array}{ll} 0, &{} {{m} \leqslant 1} \\ 1, &{} {{m} > 1.} \end{array} \right. \end{aligned}$$

With the help of particular solutions, the general solution of Eqs. (50–53) are

63 $$\begin{aligned}{}&{f_{m}}(\zeta ) = {f^{*}_{m}}(\zeta ) + {C_{1}} + {C_{2}}\exp (\zeta ) + {C_{3}}\exp (-\zeta ), \end{aligned}$$

64 $$\begin{aligned}{}&\quad \theta _{m}(\zeta ) = \theta ^{*}_{m}(\zeta ) + {C_{4}}\exp (\zeta ) + {C_{5}}\exp (-\zeta ), \end{aligned}$$

65 $$\begin{aligned}{}&\quad \phi _{m}(\zeta ) = \phi ^{*}_{m}(\zeta ) + {C_{6}}\exp (\zeta ) + {C_{7}}\exp (-\zeta ), \end{aligned}$$

66 $$\begin{aligned}{}&\quad \chi _{m}(\zeta ) = \chi ^{*}_{m}(\zeta ) + {C_{8}}\exp (\zeta ) + {C_{9}}\exp (-\zeta ). \end{aligned}$$

### Validation of the current work

Solution accuracy is validated by comparing the solution with the published work. The present work in Table 1 shows the nice agreement with the published literature^54^.

**Table 1:** Comparison of the current work.

Needle size *a*	Published results^54^	Present results
0.10	1.2888012	1.2888011
0.01	8.4924121	8.4924120
0.001	62.163713	62.163713

## Results and discussion

For the solution of mathematical model, homotopy analysis method (HAM) is used through MATHEMATICA. The various involved parameters are discussed for the different existing profiles like velocity, temperature, nanoparticles concentration, motile gyrotactic microorganisms concentration and entropy generation through the graphs in Figs. 2, 3, 4, 5, 6, 7, 8, 9, 10, 11, 12, 13, 14, 15, 16, 17, 18, 19, 20, 21, 22, 23, 24, 25, 26, 27, 28, 29, 30, 31, 32, 33, 34, 35, 36 and 37.

**Figure 2:**
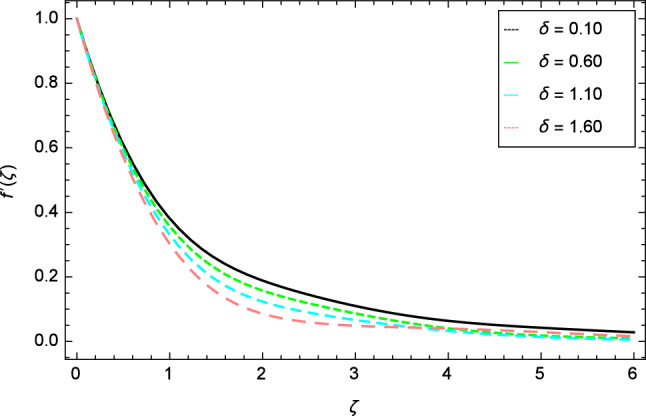
Velocity profile variation due to elasticity parameter $\delta$.

**Figure 3:**
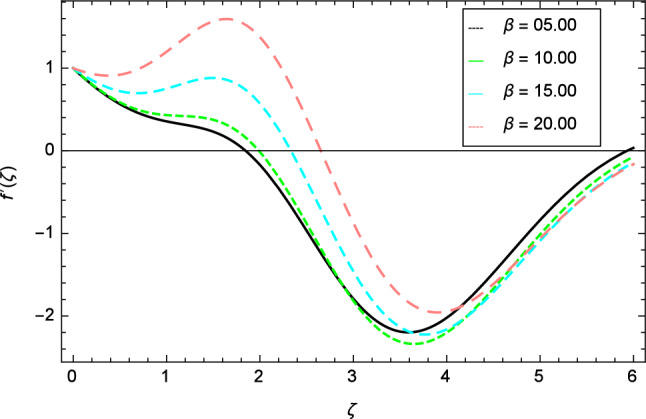
Velocity profile variation due to ferromagnetic parameter $\beta$.

### Velocity profile

**Figure 4:**
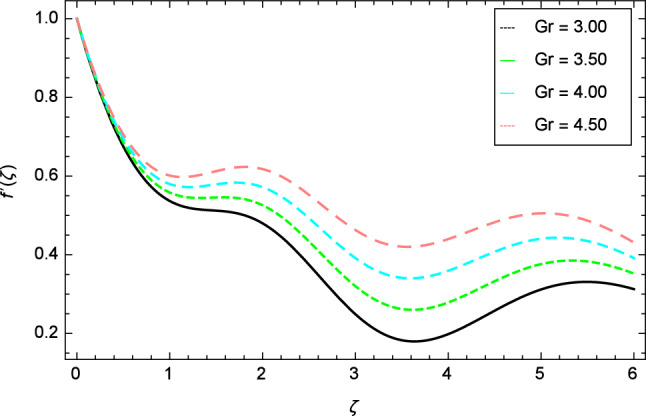
Velocity profile variation due to local Grashof number $Gr$.

**Figure 5:**
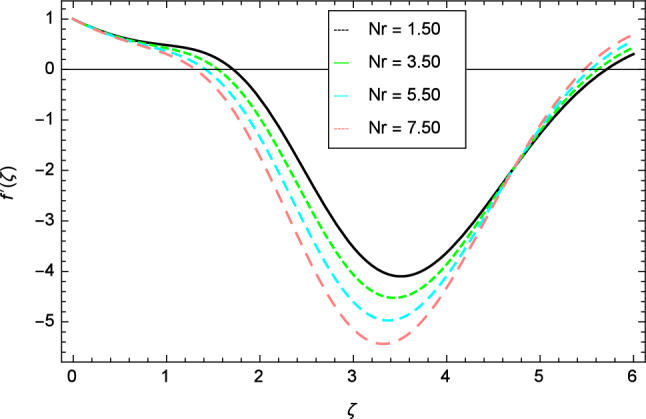
Velocity profile variation due to buoyancy ratio parameter $Nr$ .

**Figure 6:**
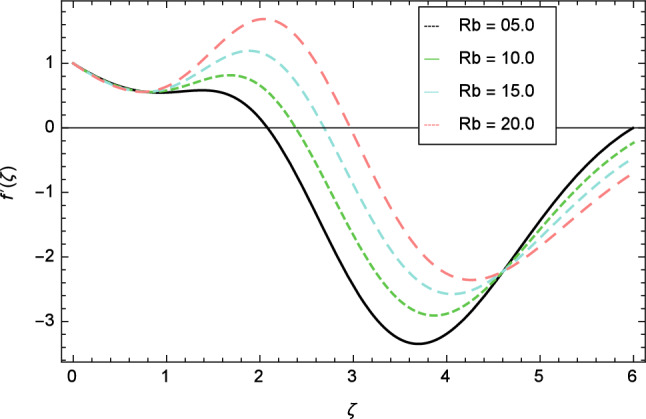
Velocity profile variation due to bioconvection Rayleigh number $Rb$.

**Figure 7:**
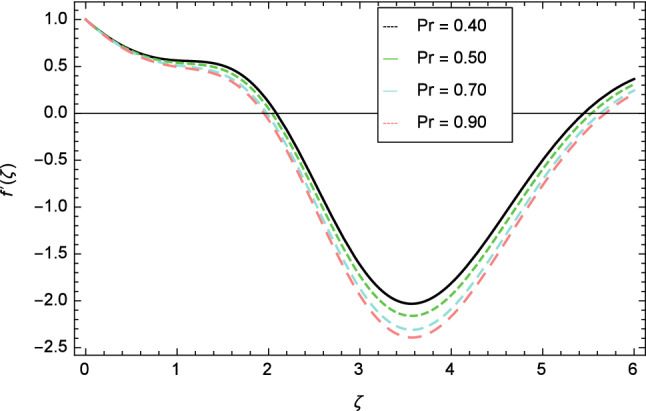
Velocity profile variation due to Prandtl number $Pr$.

**Figure 8:**
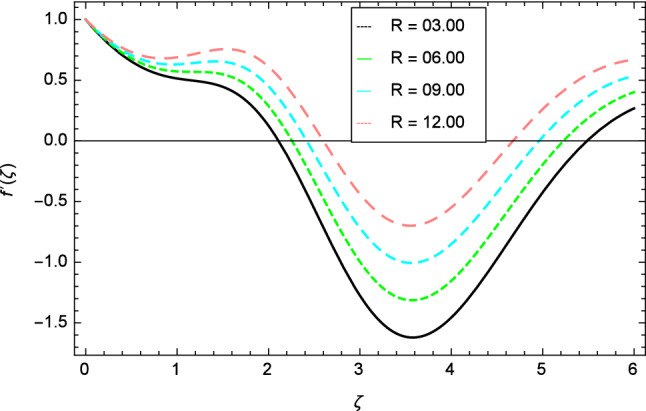
Velocity profile variation due to radiation parameter $R$.

**Figure 9:**
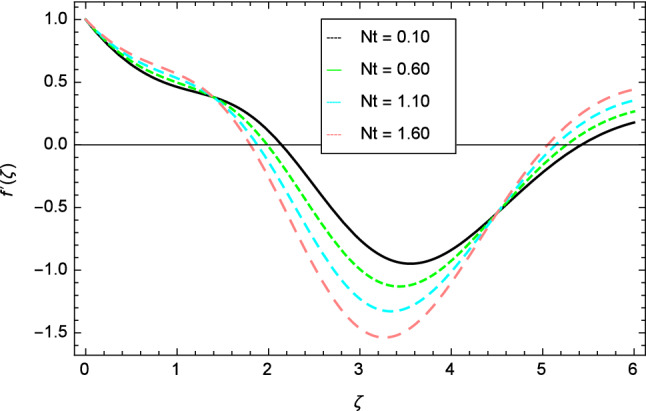
Velocity profile variation due to thermophoresis parameter $Nt$.

**Figure 10:**
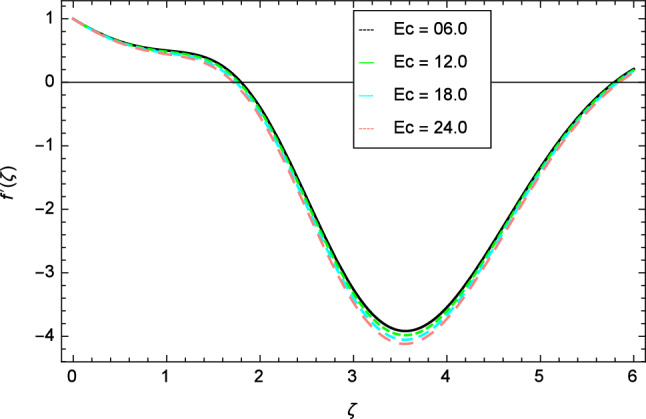
Velocity profile variation due to Eckert number $Ec$.

**Figure 11:**
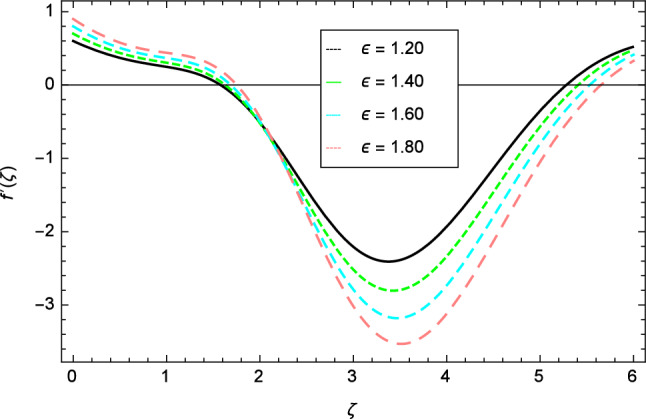
Velocity profile variation due to Curie temperature $\epsilon$.

The variation of velocity profile for distinct values of different parameters are discussed in Figs. 2, 3, 4, 5, 6, 7, 8, 9, 10 and 11. Figure 2 portrays that the velocity profile of the nanofluid decreases with the enrichment of elasticity parameter $\delta$. In the fluid motion, the resistance is so small because when the elasticity parameter $\delta$ is increased, the viscosity is depressed therefore the velocity increases. Figure 3 illustrates the behavior of ferromagnetic parameter $\beta$ over the velocity profile. It shows that with the rise of ferromagnetic parameter $\beta$, the velocity profile upsurges. It is due to rising of momentum boundary layer and physically when the ferromagnetic parameter $\beta$ is enhanced in the fluid motion. The scenary of velocity profile for local Grashof number $Gr$ is seen in Fig. 4 which shows the increment in nanofluid velocity for varying values of local Grashof number $Gr$. Actually, the local Grashof number $Gr$ is connected with the buoyancy forces. The buoyancy forces become stronger with the increase of local Grashof number $Gr$ that’s why the velocity shows increasing performance. In Fig. 5, the decrement in the velocity profile is noted for lager values of buoyancy ratio parameter $Nr$. It is due to the fact that with the enhancement of buoyancy ratio parameter $Nr$, the nanofluid particles start moving into surface of the needle therefore the fluid velocity decreases. The change in nanofluid velocity for bioconvection Rayleigh number $Rb$ is exhibited in Fig. 6. It is noted that when bioconvection Rayleigh number $Rb$ becomes larger then increasing trend is observed for nanofluid velocity profile because of the microorganisms which drag the fluid. The velocity profile against Prandtl number $Pr$ is displayed in Fig. 7. It is perceived that the Prandtl number decreases the velocity profile of nanofluid. According to the definition of Prandtl number, it is the ratio of momentum diffusivity to thermal diffusivity. When the Prandtl number $Pr$ increases then the momentum diffusivity is dominant and momentum boundary layer is decreased therefore the velocity of the nanofluid decreases. The influence of radiation parameter $R$ for velocity profile is discussed in Fig. 8. The decreasing performance is appeared in velocity profile for higher values of radiation parameter $R$. The effect of thermophoresis parameter $Nt$ on the velocity profile is indicated in Fig. 9. The intensifying values of thermophoresis parameter $Nt$ cause to increase the velocity profile of the fluid. Figure 10 presents the graphical role of velocity profile for growing values of Eckert number $Ec$. The rise in Eckert number $Ec$, reduces the nanofluid velocity profile. This is due to the fact that kinetic energy of the system is transformed into the heat energy because of viscous dissipation, which causes the reduction in the velocity profile. The deviation of Curie temperature parameter $\epsilon$ for velocity profile is inspected in Fig. 11. The reduction for nanofluid velocity profile is observed for expanding values of Curie temperature parameter $\epsilon$.

### Temperature profile

**Figure 12:**
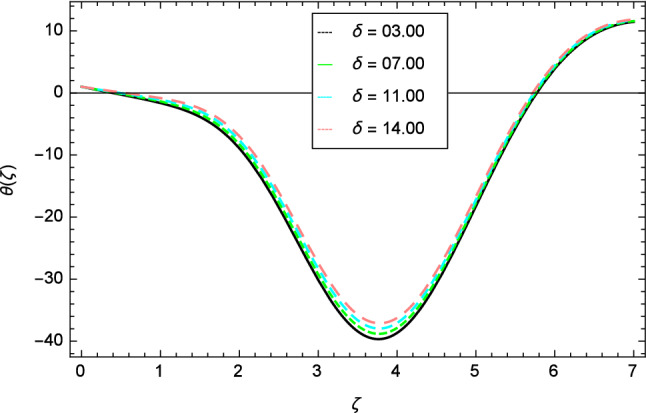
Temperature profile variation due to elasticity parameter $\delta$.

**Figure 13:**
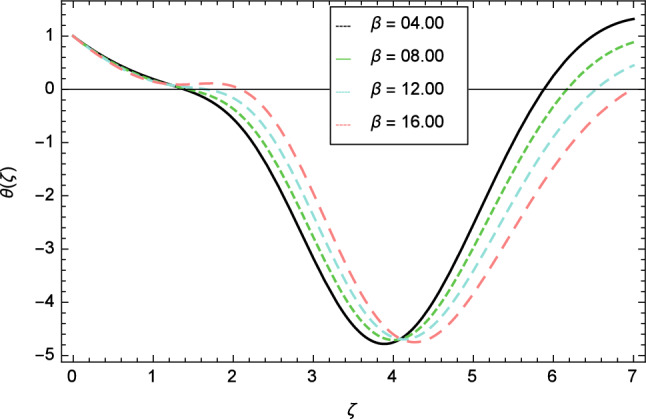
Temperature profile variation due to ferromagnetic parameter $\beta$.

**Figure 14:**
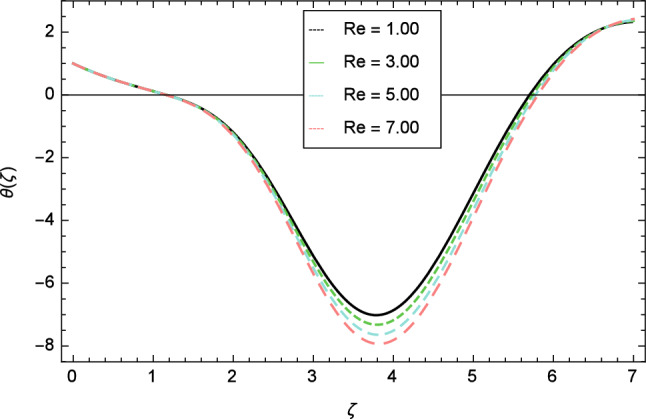
Temperature profile variation due to Reynolds number $Re$.

**Figure 15:**
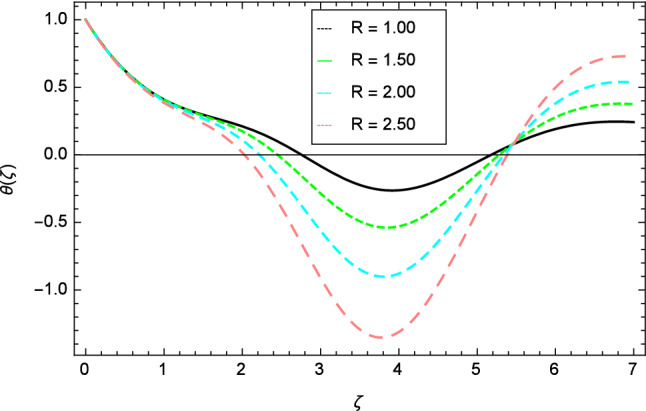
Temperature profile variation due to radiation parameter $R$.

**Figure 16:**
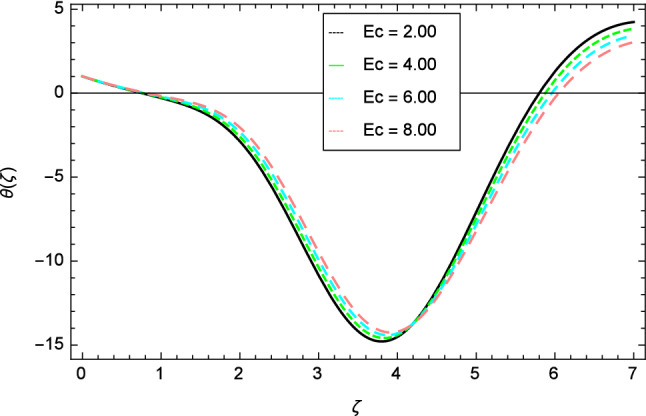
Temperature profile variation due to Eckert number $Ec$ .

Figures 12, 13, 14, 15 and 16 describe the influence of different parameters on the temperature profile. From Fig. 12, it is detected that nanofluid temperature profile rises with the intensification of elasticity parameter $\delta$. Figure 13 represents the graphical behavior of fluid temperature for ferromagnetic parameter $\beta$. In this Fig. 13, the temperature profile falls due to the augmentation of ferromagnetic parameter $\beta$. The effect of Reynolds number $Re$ on the temperature profile is shown in Fig. 14. It is viewed that with the expansion of Reynolds number $Re$, it leads to hike the nanofluid temperature. Figure 15 shows that with the inflation of radiation parameter $R$, temperature profile of the fluid is in declining behavior. Physically, when the radiation parameter increases, the Rosseland radiative absorption coefficient $k_{e}$ declines as defined by the expression for $R =\frac{16\sigma _{1}{T_{\infty }^{3}}}{3k_{e}K}$. Therefore the heat flux diminishes. That’s why the nanofluid temperature decreases. Figure 16 visualized the impact of Eckert number $Ec$ on the nanofluid temperature profile. In this graph, it is observed that the larger values of Eckert number $Ec$ enhance the temperature profile. The thermal boundary layer thickness of the nanoparticles and transportation energy are surged due to the enhancement of Eckert number $Ec$.

**Figure 17:**
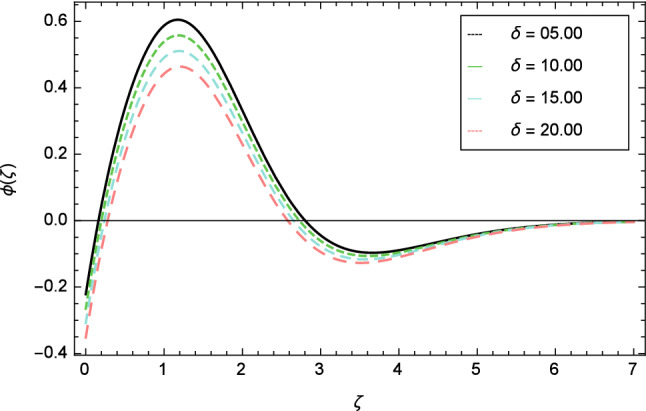
Nanoparticles concentration profile variation due to elasticity parameter $\delta$.

**Figure 18:**
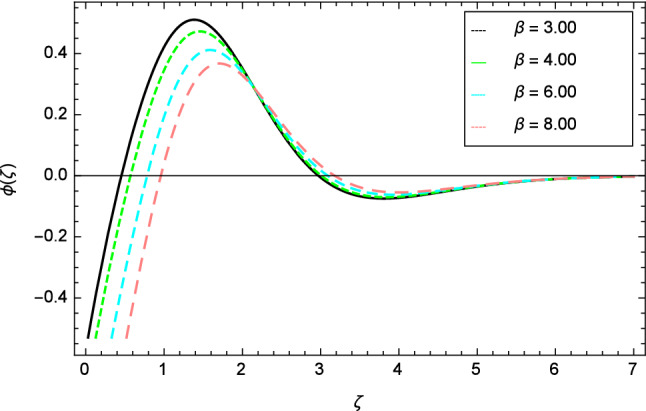
Nanoparticles concentration profile variation due to ferromagnetic parameter $\beta$.

### Nanoparticles concentration profile

**Figure 19:**
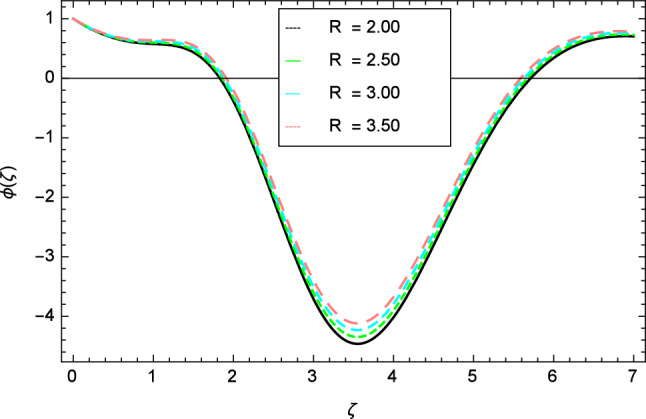
Nanoparticles concentration profile variation due to radiation parameter $R$.

**Figure 20:**
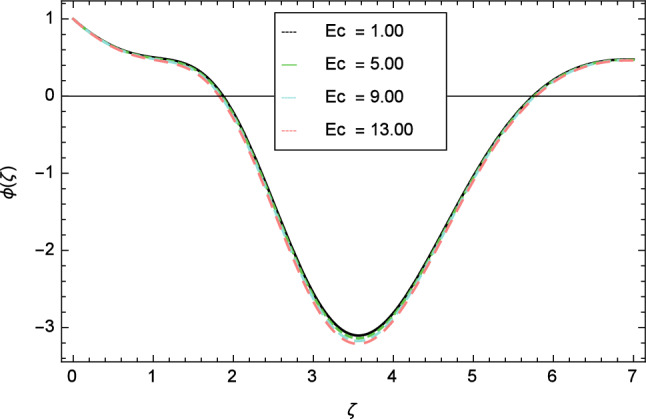
Nanoparticles concentration profile variation due to Eckert number $Ec$.

**Figure 21:**
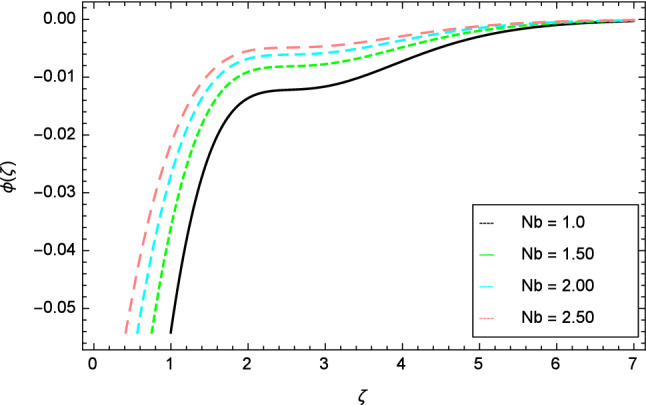
Nanoparticles concentration profile variation due to Brownian motion parameter $Nb$.

**Figure 22:**
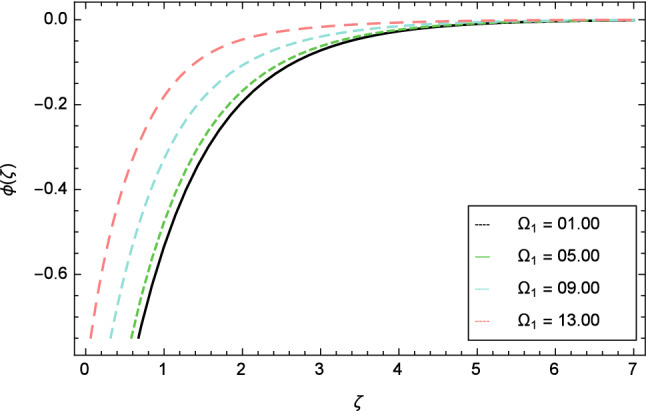
Nanoparticles concentration profile variation due to temperature difference parameter $\Omega _{1}$.

**Figure 23:**
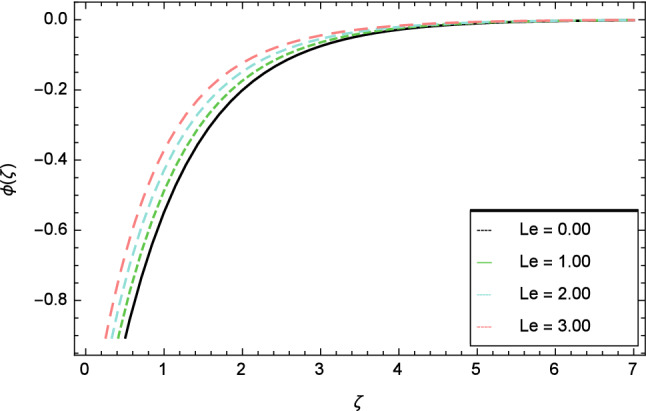
Nanoparticles concentration profile variation due to Lewis number $Le$.

**Figure 24:**
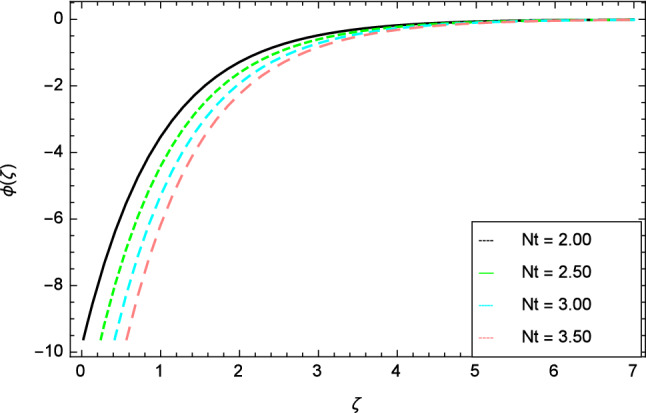
Nanoparticles concentration profile variation due to thermophoresis parameter $Nt$.

In this section, nanoparticles concentration profile is sketched for various parameters. Figure 17 deals with nanoparticles concentration profile against elasticity parameter $\delta$. It is remarked that with the improvement of elasticity parameter $\delta$, the nanoparticles concentration profile is reduced. Figure 18 is designated to show that by the increment of ferromagnetic parameter $\beta$, lessening in nanoparticles concentration profile is noted. Elevation in fluid temperature is studied in Fig. 19 for radiation parameter $R$. The illustration of Eckert number $Ec$ and the nanoparticles concentration profile are shown in Fig. 20. Increments in Eckert number $Ec$ decrease the nanoparticles concentration profile. The nanoparticles concentration profile verses Brownian motion parameter $Nb$ is exhibited in Fig. 21. It is revealed that, the enhancing behavior in nanoparticles concentration profile is notable for various values of Brownian motion parameter $Nb$. The random motion of the fluid particles become larger due to the enhancement of Brownian motion parameter $Nb$ therefore the nanoparticles concentration show variation in the profile. It is evident from Fig. 22 that increment in nanoparticles concentration profile is exposed for fluctuating values of temperature difference parameter $\Omega _{1}$. Figures 23 and 24 are prepared to observe the influence of Lewis number $Le$ and thermophoresis parameter $Nt$ on the nanoparticles concentration profile. The Lewis number $Le$ increases the nanoparticles concentration profile but opposite trend is seen in nanoparticles concentration profile for thermophoresis parameter $Nt$. Physically, the Lewis number is stated as the ratio of thermal diffusivity to Brownian diffusion and when Brownian diffusivity is smaller then the nanoparticles fraction increases. As the thermal conductivity of the nanofluid increases, it infiltrates deeper into nanoparticles by the increase of thermophoresis parameter $Nt$ and finally reduces the thickness of the concentration boundary layer. As a result, increasing the thermophoresis parameter $Nt$ diminishes the nanoparticles concentration profile. Also, the random motion of the fluid particles decreases.

### Motile gyrotactic microorganism profile

**Figure 25:**
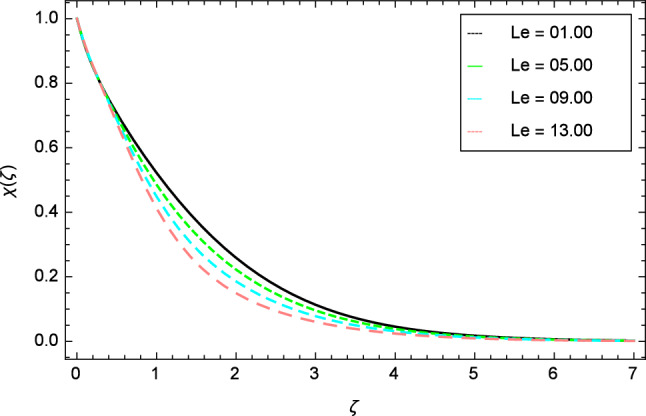
Motile gyrotactic microorganisms profile variation due to Lewis number $Le$.

**Figure 26:**
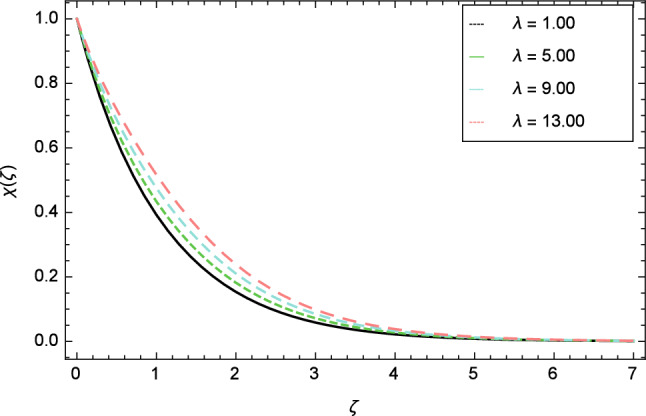
Motile gyrotactic microorganisms profile variation due to bioconvection constant parameter $\lambda$.

**Figure 27:**
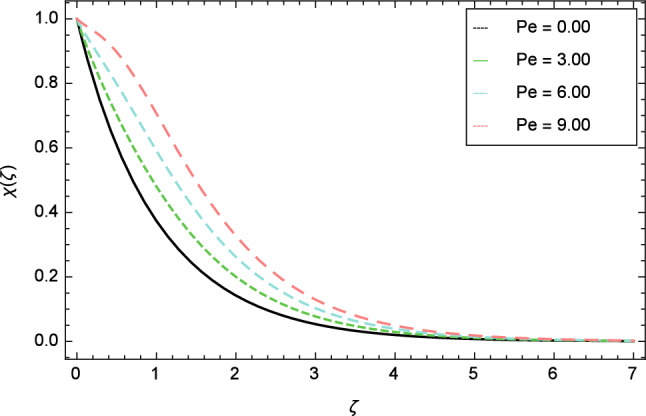
Motile gyrotactic microorganisms profile variation due to Peclet number $Pe$.

**Figure 28:**
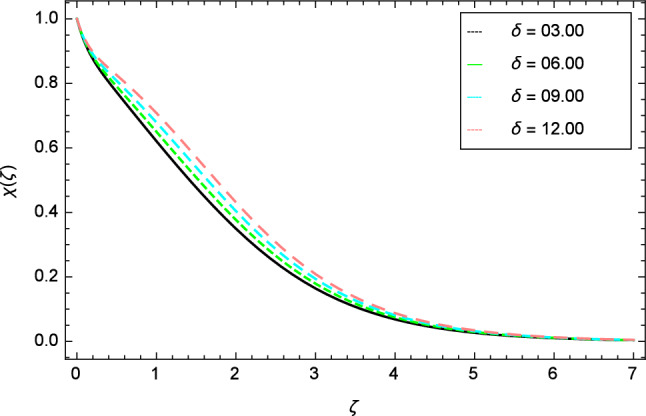
Motile gyrotactic microorganisms profile variation due to elasticity parameter $\delta$.

**Figure 29:**
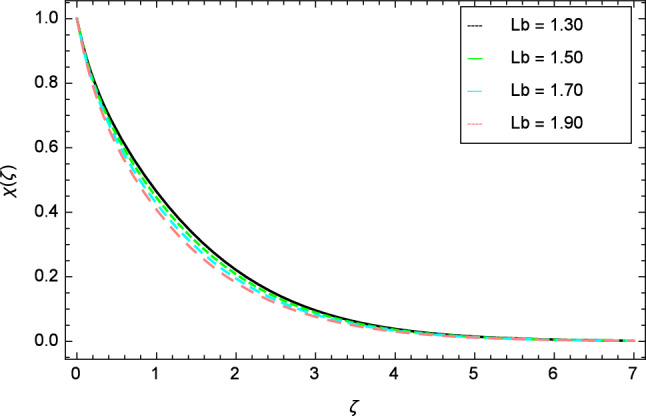
Motile microorganisms profile variation due to bioconvection Lewis number $Lb$.

Figures 25, 26, 27, 28 and 29 are plotted for motile gyrotactic microorganisms through the variation of different parameters. Figure 25 depicts that when the Lewis number $Le$ increases, motile gyrotactic microorganisms concentration is decreased because the Lewis number $Le$ and the mass diffusion are inversely proportional to each other. Figure 26 is drawn for the bioconvection constant parameter $\lambda$ on the motile gyrotactic microorganisms. It is found that with the enlargement of bioconvection constant parameter $\lambda$, the amplification in motile gyrotactic microorganisms motion is discovered. Figure 27 scrutinizes influence of bioconvection Peclet number $Pe$ on motile gyrotactic microorganisms. It is clear from this figure that increasing values of Peclet number $Pe$ increase the flow of motile gyrotactic microorganisms. The most important thing is that the variation in motile gyrotactic microorganisms profile is more dominant in case of bioconvection Peclet number $Pe$ and bioconvection Lewis number $Lb$. The consequence of elasticity parameter $\delta$ and the motile gyrotactic microorganisms profile is deliberated in Fig. 28. The higher values of elasticity parameter $\delta$ leads to grown up the nanofluid motile gyrotactic microorganisms profile. Figure 29 demonstrates the relation between the motile gyrotactic microorganisms profile and the bioconvection Lewis number $Lb$.

It is perceived that the dwindle in motile gyrotactic microorganism profile is reflected for growing values of bioconvection Lewis number $Lb$. Physically it is seen that enhancing the numerical values of bioconvection Lewis number $Lb$ leads to decrease the motile gyrotactic microorganisms profile.

### Entropy generation profile

**Figure 30:**
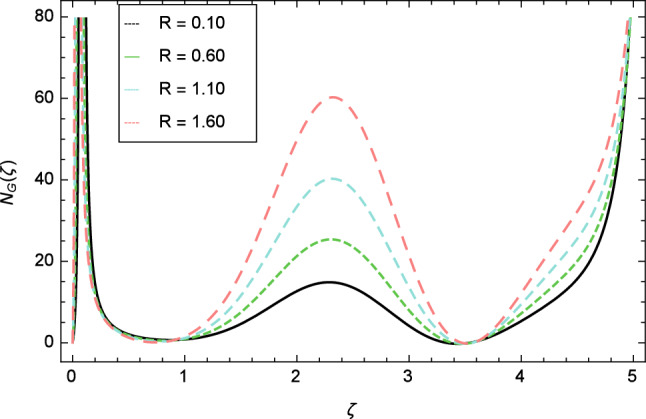
Entropy generation profile variation due to radiation parameter $R$.

**Figure 31:**
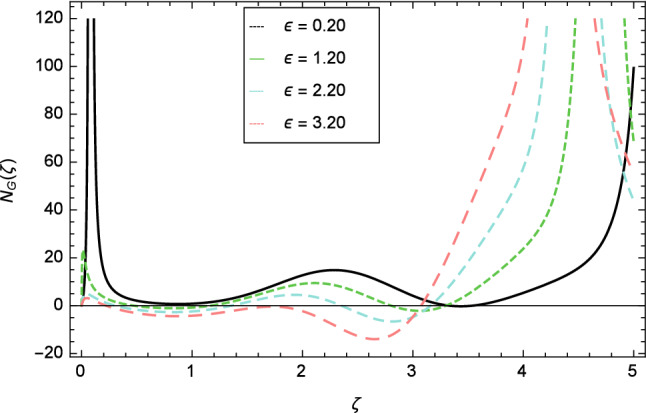
Entropy generation profile variation due to Curie temperature $\epsilon$.

**Figure 32:**
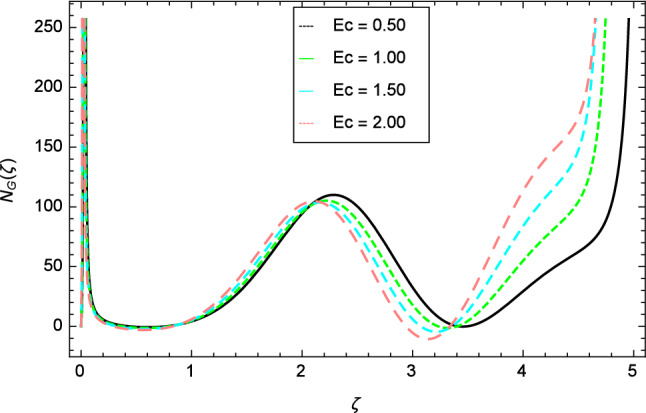
Entropy generation profile variation due to Eckert number $Ec$.

**Figure 33:**
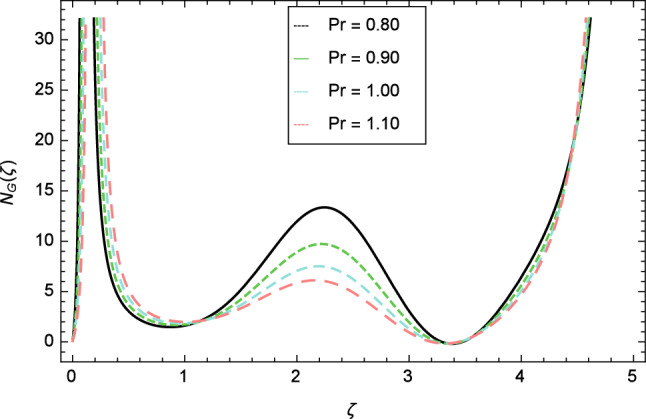
Entropy generation profile variation due to Prandtl number $Pr$.

**Figure 34:**
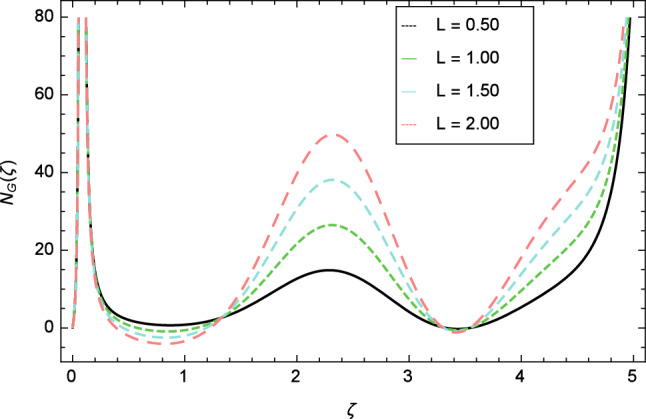
Entropy generation profile variation due to diffusive parameter $L$.

**Figure 35:**
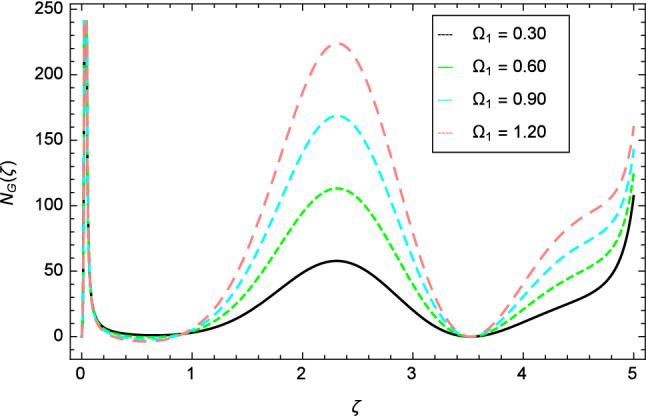
Entropy generation profile variation due to temperature difference parameter $\Omega _{1}$.

**Figure 36:**
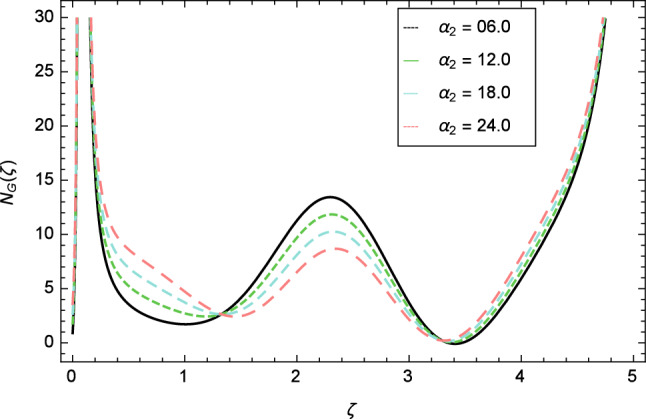
Entropy generation profile variation due to concentration difference parameter ${\alpha _{2}}$.

Figure 30 displays the effect of radiation parameter $R$ on entropy generation and found that upswing in entropy generation profile is appeared for high values of radiation parameter $R$. This is all because of the heightened energy system owing to the larger estimates of $R$. Intensification in entropy profile is noted in Fig. 31 for Curie temperature $\epsilon$. From Fig. 32, it is seen that increment in entropy graph is exist for larger values of Eckert number $Ec$. Since heat is a form of disorganized energy (energy with less quality and more entropy) therefore with increasing Eckert number, the kinetic energy of fluid (high-grade energy) is converted into heat energy (low-grade energy) and consequently entropy generation increases. Figure 33 exhibits the variance of entropy for upgrading values of Prandtl number $Pr$. The entropy profile of the nanofluid is decayed for Prandtl number $Pr$. The entropy generation profile is boost up as shown in Figs. 34 and 35 for various values of diffusive parameter $L$ and temperature difference parameter $\Omega _{1}$. Figure 36 explicates the significance of entropy generation profile for nanoparticles concentration difference parameter $\alpha _{2}$.

**Figure 37:**
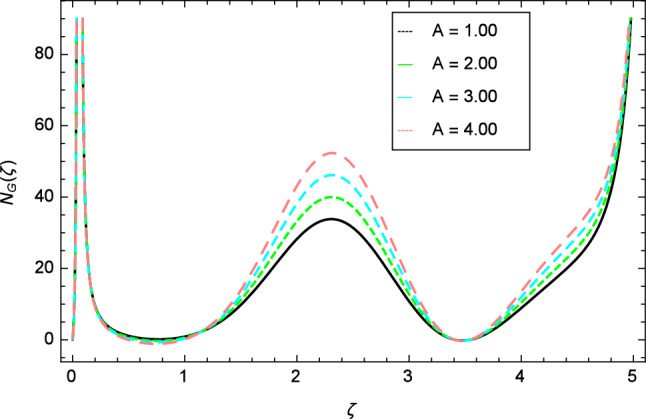
Entropy generation profile variation due to dimensionless constant parameter ${A }$.

With the expansion of nanoparticles concentration difference parameter $\alpha _{2}$, the entropy generation profile is reduced. In Fig. 37, for larger values of dimensionless constant parameter $A$, there is a high inflation of the entropy generation profile.

## Conclusions

In the present analysis, the problem of two-dimensional non-Newtonian second grade nanofluid with magnetic dipole effect and gyrotactic microorganism past a thin needle is performed. The effects of Arrhenius activation energy and binary chemical reaction are also detected with the analysis of entropy generation. The solution of the model is obtained by using the most powerful analytical method called the homotopy analysis method (HAM) using MATHEMATICA. Over the heat and mass transfer second-grade nanofluid flow, the effects of different physical parameters are determined. The following results are summarised as

1. The velocity of the nanofluid increases when the ferromagnetic parameter $\beta$, local Grashof number $Gr$, bioconvection Rayleigh number $Rb$ and radiation parameter $R$ are increased.
2. Retardation in nanofluid velocity profile is noted for higher values of elasticity parameter $\delta$, buoyancy ratio parameter $Nr$, Prandtl number $Pr$, thermophoresis parameter $Nt$, Eckert number $Ec$ and Curie temperature parameter $\epsilon$.
3. The elasticity parameter $\delta$ increases the temperature of the nanofluid.
4. Fluid temperature reduces for various values of Reynolds number $Re$, radiation parameter $R$, and Eckert number $Ec$.
5. Increasing behavior of nanoparticles concentrtaion profile is found for radiation parameter $R$, Brownian motion parameter $Nb$, temperature difference parameter $\Omega _{1}$, Lewis number $Le$ and declining reaction of nanopartilces concentration is observed for elasticity parameter $\delta$, ferromagnetic parameter $\beta$, Eckert number $Ec$ and thermophoresis parameter $Nt$.
6. The influence of elasticity parameter $\delta$, bioconvection Peclet number $Pe$, Eckert number $Ec$, thermophoresis parameter $Nt$ and bioconvection constant parameter $\lambda$ intensify the gyrotactic motile microorganisms profile.
7. The Lewis number $Le$ and bioconvection Lewis number $Lb$ have decreasing effects on the motile gyrotactic microorganisms profile.
8. The nanofluid entropy generation is enhanced with the increasing of radiation parameter $R$, Eckert number $Ec$, Lewis number $Le$, temperature difference parameter $\Omega _{1}$ and dimensionless constant parameter $A$ and is reduced for Curie temperature $\epsilon$, Prandtl number $Pr$ and nanoparticles concentration difference parameter $\alpha _{2}$.

## Supplementary Information

Supplementary Information 1.

## Data Availability

Data is available upon reasonable request to corresponding author.

## Nomenclature

$$M$$: Magnetization
$$(u , v )$$: Velocity components
$$\mu$$: Dynamic viscosity
$$\nu$$: Kinematic viscosity
$$\psi$$: Stream function
$$(x , r )$$: Cylindrical coordinates
$R_{1}$: Radius of thin needle
$$\alpha$$: Thermal diffusivity
$\alpha _{1}$: Second grade fluid parameter
$$a$$: Needle size
$\tau$: Nanofluid effective heat capacity ratio
$$\rho$$: Density
${\beta ^{*}}$: Coefficient of volumetric volume expansion
$$\mu _{0}$$: Magnetic permeability
$$H$$: Magnetic field
$$\hbox {f}$$: Base fluid
$$\hbox {P}$$: Fluid pressure
$W _{c}$: Maximum amount of swimming cell
${c}_{1}$: Distance between origin and center of magnetic dipole
$T _{c_{1}}$: Dimensional Curie temperature
$\rho _{f}$: Density of base fluid
$\rho _{P}$: Density of nanoparticles
$\rho _{m}$: Density of motile microorganism
$U _{w}$: Velocity at the wall
$T _{w}$: Temperature at the wall
$C _{w}$: Nanoparticles concentration at the wall
$N _{w}$: Motile gyrotactic microorganism concentration at the wall
$$T _{\infty }$$: Ambient temperature
$C _{\infty }$: Ambient nanoparticles concentration
$N _{\infty }$: Ambient motile gyrotactic microorganisms concentration
$$T$$: Temperature
$$C$$: Nanoparticles concentration
$N$: Gyrotactic microorganism distribution
$$g$$: Gravitational acceleration
$D _{B}$: Brownian diffusion coefficient of nanoparticles
$D _{T}$: Thermophoresis diffusion coefficient
$K$: Nanofluid thermal conductivity
$C _{P}$: Specific heat at constant pressure
$q_{r}$: Radiative heat flux
$k_{e}$: Mean absorption coefficient
$\sigma _{1}$: Stefan–Botlzmann constant
$n$: Fitted rate constant
$$E_{a_{1}}$$: Activation energy
$a_{1}$: Positive dimensional constant
*E*: Non-dimensional activation energy parameter
$$k _{cr}$$: Chemical reaction
$D_{m}$: Brownian diffusion coefficient of microorganisms
$$\zeta$$: Similarity variable
$$f^{\prime }$$($$\zeta$$): Dimensionless velocity
$$\theta$$($$\zeta$$): Dimensionless temperature
$\phi$($\zeta$): Dimensionless nanoparticles concentration
$\chi$($\zeta$): Dimensionless motile gyrotactic microorganisms concentration
$$\delta$$: Elasticity parameter
${\gamma _{1}}$: Distance between origin and center of the magnetic dipole
${\gamma }$: Strength of magnetic field
$$k^{*}$$: Pyromagnetic coefficient
$$\beta$$: Ferromagnetic parameter
$$Ec$$: Eckert number
$\epsilon$: Dimensionless Curie temperature ratio
$Gr$: Local Grashof number
$Nr$: Buoyancy ratio parameter
$Rb$: Bioconvection Rayleigh number
$$R$$: Radiation parameter
$$Pr$$: Prandtl number
$Nb$: Brownian motion parameter
$$Nt$$: Thermophoresis parameter
$$Le$$: Lewis number
$\Gamma$: Binary chemical reaction parameter
$\Omega _{1}$: Temperature differnce parameter
$Lb$: Bioconvection Lewis number
$Pe$: Bioconvection Peclet number
$\lambda$: Bioconvection constant parameter
$$Re$$: Reynolds number
$$E^{\prime \prime \prime }_{gen}$$: Entropy generation
$E^{\prime \prime \prime }_{0}$: Characteristics entropy generation
$N_{G}(\zeta )$: Dimensionless entropy generation rate
$$L$$: Diffusive parameter
${A }$: Dimensionless constant parameter
${\alpha _{2}}$: Nanoparticles concentration difference parameter
${\prime }$: Differentiation with respect to $\zeta$
