# Supplementary material for: Mechanical analysis of non-Newtonian nanofluid past a thin needle with dipole effect and entropic characteristics
Source: Sci Rep. 2021 Sep 29;11:19378. doi: 10.1038/s41598-021-98128-z (PMC8481475; doi:10.1038/s41598-021-98128-z)
Supplement: Supplementary file 1 — Supplementary Information 1. [file 41598_2021_98128_MOESM1_ESM.pdf]

Appendix

The magnetic dipole is directed from the surface at the center c_1 and its center lies over the $r-axis$. In the positive $x-direction$, the magnetic field H influences the ferrofluid since the magnetic dipole impact is discussed. The scalar magnetic potential expression is [50, 51]

$$\Omega^* = \frac{\gamma}{2\pi}\frac{x}{x^2+(r+c_1)^2}, \tag{1}$$

where c_1 represents the distance between origin and center of magnetic dipole and γ is strength of magnetic field. In components form the scaler magnetic potential are [50, 51]

$$\frac{\partial H}{\partial x} = -\frac{\partial \Omega^*}{\partial x} = \frac{\gamma}{2\pi}\frac{x^2-(r+c_1)^2}{\left(x^2+(r+c_1)^2\right)^2}, \tag{2}$$

$$\frac{\partial H}{\partial r} = -\frac{\partial \Omega^*}{\partial r} = \frac{\gamma}{2\pi}\frac{2x(r+c_1)}{\left(x^2+(r+c_1)^2\right)^2}. \tag{3}$$

In this case the magnetic body force is proportional to the square root of the absolute of the magnetic field [50,51] as

$$H = \sqrt{\left(\frac{\partial \Omega^*}{\partial x}\right)^2 + \left(\frac{\partial \Omega^*}{\partial r}\right)^2}. \tag{4}$$

In component form

$$\frac{\partial H}{\partial x} = -\frac{\gamma}{2\pi}\frac{2x}{(r+c_1)^4}, \tag{6}$$

$$\frac{\partial H}{\partial r} = -\frac{\gamma}{2\pi}\left(\frac{-2}{(r+c_1)^3} + \frac{4x}{(r+c_1)^5}\right). \tag{7}$$

The variance of magnetization M as a function of temperature is defined below

$$M = k^*\left(T_{c_1} - T\right), \tag{8}$$

where pyromagnetic coefficient is designed by k^* and the dimensional Curie temperature is denoted by T_{c_1} .
